# Gene Expression has Distinct Associations with Brain Structure and Function in Major Depressive Disorder

**DOI:** 10.1002/advs.202205486

**Published:** 2023-01-13

**Authors:** Shu Liu, Abdel Abdellaoui, Karin J. H. Verweij, Guido A. van Wingen

**Affiliations:** ^1^ Amsterdam UMC location University of Amsterdam Department of Psychiatry Amsterdam Neuroscience, Amsterdam Meibergdreef 5 Amsterdam 1100 DD The Netherlands

**Keywords:** brain function, brain structure, gene expression, major depressive disorder

## Abstract

Major depressive disorder (MDD) is associated with structural and functional brain abnormalities. MDD as well as brain anatomy and function are influenced by genetic factors, but the role of gene expression remains unclear. Here, this work investigates how cortical gene expression contributes to structural and functional brain abnormalities in MDD. This work compares the gray matter volume and resting‐state functional measures in a Chinese sample of 848 MDD patients and 749 healthy controls, and these case‐control differences are then associated with cortical variation of gene expression. While whole gene expression is positively associated with structural abnormalities, it is negatively associated with functional abnormalities. This work observes the relationships of expression levels with brain abnormalities for individual genes, and found that transcriptional correlates of brain structure and function show opposite relations with gene dysregulation in postmortem cortical tissue from MDD patients. This work further identifies genes that are positively or negatively related to structural abnormalities as well as functional abnormalities. The MDD‐related genes are enriched for brain tissue, cortical cells, and biological pathways. These findings suggest that distinct genetic mechanisms underlie structural and functional brain abnormalities in MDD, and highlight the importance of cortical gene expression for the development of cortical abnormalities.

## Graphical abstract

This paper investigates brain abnormalities in MDD, and observes opposite relationships of structural and functional abnormalities with gene expression in both Asian and European populations. Moreover, the transcriptional correlates of brain abnormalities show significant correlations with gene dysregulation in postmortem tissue samples from MDD patients. These findings suggest that distinct genetic mechanisms underlie brain abnormalities.

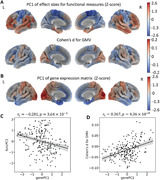

## 1 Introduction

Major depressive disorder (MDD) is a highly prevalent psychiatric disorder^[^ ^1^ ^]^ that is the third leading cause of disability worldwide.^[^ ^2^ ^]^ Despite decades of research, the pathophysiology of MDD is not well understood. The etiology of MDD is complex and influenced by genetic factors. Twin studies have estimated that the heritability of MDD is ≈ 37%,^[^ ^3^ ^]^ and genome‐wide association studies **(GWASs)** have captured ≈9% of the heritability with common single nucleotide polymorphisms **(SNPs)**.^[^ ^4^ ^]^ Moreover, MDD is highly polygenic, resulting from the joint effects of many genetic variants with small effects scattered across the genome.^[^ ^5^ ^]^

Previous research has shown that certain brain regions are affected in MDD individuals. There are structural and functional differences between depressed individuals and healthy controls in various brain regions (e.g., the anterior cingulate and frontal regions).^[^ ^6^ ^]^ To investigate the neural mechanisms of genetic risk for MDD, neuroimaging intermediate phenotypes can be used to link genetic variation to this complex psychiatric syndrome.^[^ ^7^ ^]^ For example, the genetic predisposition to MDD is associated with brain structure in the orbitofrontal cortex^[^ ^8^ ^]^ and brain function in the lateral frontal and precentral regions.^[^ ^9^ ^]^ Although imaging genetics analyses for MDD have provided evidence that genetic factors play a major role in these brain phenotypes implicated in depression, the intermediate processes remain to be elucidated.

Gene expression is the most fundamental molecular process through which genes can regulate the differentiation, development, and functioning of brain cells and tissues.^[^ ^10^ ^]^ Expression levels of genome‐wide genes have been estimated in post‐mortem brain tissue from six donors, and have been made public as the Allen Human Brain Atlas (AHBA)^[^ ^11^ ^]^ and this information has been widely used to examine the transcriptional correlates of brain structure and function.^[^ ^12^ ^]^ Brain‐wide gene expression data has been associated with cortical volume,^[^ ^13^ ^]^ MRI signal intensity,^[^ ^14^ ^]^ and resting‐state activity.^[^ ^15^ ^]^ Previous studies that combined neuroimaging and AHBA gene expression data identified several genes related to structural brain abnormalities in various psychiatric disorders.^[^ ^16^ ^]^ A previous study has linked depression‐related cortical abnormalities to normative patterns of gene expression^[^ ^17^ ^]^ in healthy individuals with negative affect measures and individuals who have experienced depressive illness in the past. Another study by Li et al.^[^ ^18^ ^]^ found a positive correlation between gene expression and structural brain abnormalities of morphometric similarity in patients with depression. However, this study used a relatively small sample size which could have led to insufficient statistical power to detect important associations, and the study only focused on structural brain abnormalities and did not consider functional brain abnormalities.

The aim of this study was to extend the work by Li et al.^[^ ^18^ ^]^ by substantially increasing the sample size and by including brain function. We used T1 and resting‐state functional MRI data from 848 MDD patients and 794 healthy controls to compare gray matter volume (GMV) and three major functional measures in 210 cortical brain regions based on Brainnetome (BN) atlas.^[^ ^19^ ^]^ We then linked the expression level of 15 633 genes in these regions to the identified case‐control structural and functional differences. The UK Biobank dataset was used to replicate the transcriptome‐neuroimaging relationships. We further investigated the relationships of transcriptional correlates of in vivo brain abnormalities with ex vivo gene dysregulation for psychiatric disorders. Moreover, we selected genes transcriptionally involved in structural or functional brain differences, and performed gene set enrichment analysis for tissues, cortical cell types, and biological pathways to understand the functional annotations of identified gene sets.

## 2 Results

### 2.1 Case‐Control Brain Differences

**Figure 1:**
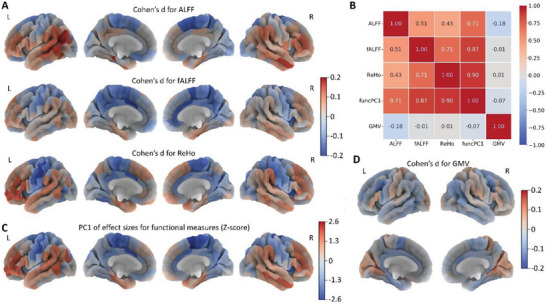
Case‐control structural and functional brain differences for 210 cortical regions. A) Differences (Cohen's d) for functional brain measures including amplitude of low‐frequency fluctuation (ALFF), fractional ALFF (fALFF), and regional homogeneity (ReHo); B) the correlations between case‐control differences; C) the first principle component of effect sizes for three functional measures (funcPC1) (Z‐score normalization); D) differences (Cohen's d) for gray matter volume (GMV).

Using the REST‐meta‐MDD dataset with 848 MDD patients (534 females and 314 males, age ranges: 18–65 years old) and 794 healthy controls (467 females and 327 males, age ranges: 18–64 years old), we compared the case‐control differences of three major functional brain measures (amplitude of low‐frequency fluctuation (ALFF), fractional ALFF (fALFF), and regional homogeneity (ReHo)) in 210 cortical regions. We found similar case‐control difference patterns across the brain for these three functional measures, generally with increased neuronal activity in lateral regions and decreased activity in medial regions, although different statistically significant brain functional abnormalities were observed after false discovery rate (FDR) multiple testing correction (**Figure** **1**A, Figure S1, Supporting Information). MDD patients had significantly decreased ALFF in the left medial superior frontal cortex and significantly increased ALFF in inferior parietal cortex and temporal regions (Figure S1A, Supporting Information), whereas the MDD patients had significant decreased fALFF in the medial superior frontal and caudodorsal/pregenual cingulate cortex (Figure S1B, Supporting Information). In MDD patients, ReHo was significantly increased in the lateral prefrontal cortex, and decreased in the dorsal insula, precentral/postcentral/paracentral cortex, and caudodorsal cingulate cortex (Figure S1C, Supporting Information). The effect sizes for these functional differences ranged from Cohen's d −0.2 to 0.2, Moreover, brain‐wide case‐control differences for ALFF, fALFF, and ReHo were highly positively correlated with each other (correlation *r*_s_ ranged from 0.43 to 0.71) (Figure 1B, Figure S1D, Supporting Information). We therefore used principle component analysis (PCA) to extract the first principal component (PC) of effect sizes for functional measures, defined as funcPC1, which explained 71% variance of functional brain abnormalities in MDD (Figure 1C).

GMV showed smaller effect sizes (Cohen's d: −0.1–0.12), with decreased GMV in frontal and temporal cortex and increased GMV in occipital and parietal regions (Figure 1D), but none of these case‐control differences were significant.

To verify whether the observed case‐control differences were dependent on the brain parcellation scheme, we repeated the analyses based on the Desikan–Killiany (DK) atlas with 68 cortical regions. These results showed a comparable pattern of structural and functional differences (Figure S2, Supporting Information).

### 2.2 Relationships of Gene Expression with Case‐Control Differences

**Figure 2:**
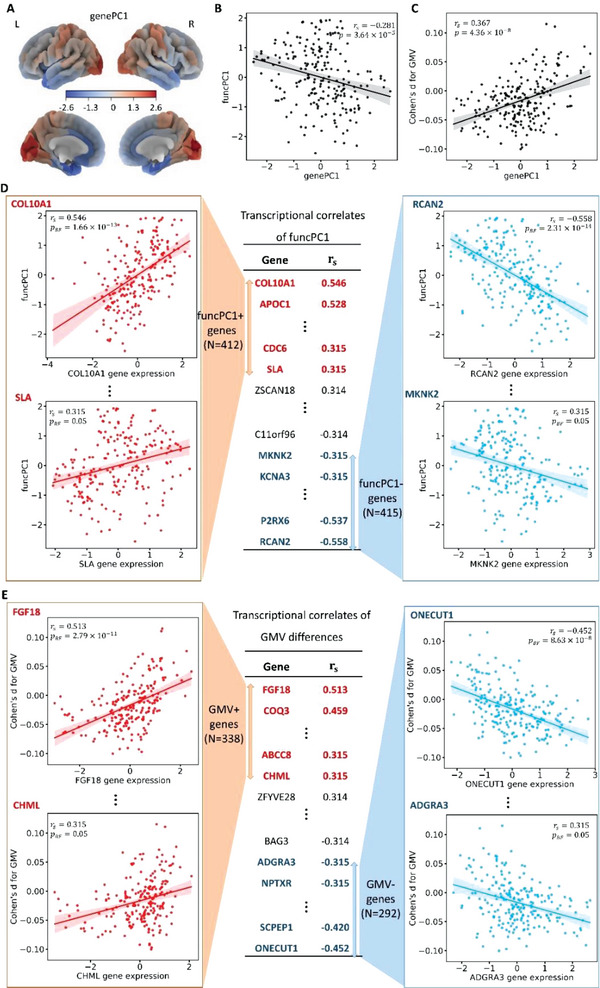
Relations of gene expression with case‐control differences for 210 cortical regions. A) The first principal component of gene expression (genePC1); B) Relationships of genePC1 with the first principal component of three major functional measures (funcPC1); C) Relationships of genePC1 with gray matter volume (GMV) differences; D) Genes positively or negatively related to funcPC1 (funcPC1+ or funcPC1− genes); E) Genes positively or negatively related to GMV differences (GMV+ or GMV− genes). *p*_BF_ indicates the p values corrected by Bonferroni multiple testing correction.

We used a whole brain transcriptomic dataset, AHBA gene expression matrix based on BN atlas (210 regions × 15 633 genes). PCA was used to extract the first PC from the matrix (genePC1), which explained 18.6% of the variance (**Figure** **2**A). We then estimated the association of genePC1 with funcPC1 as well as Cohen's d for GMV and the functional measures. GenePC1 was negatively correlated with funcPC1 (*r*_s_ = −0.281, *p* = 3.64 × 10^−5^) (Figure 2B), and genePC1 consistently showed negative correlation with effect sizes for ALFF, fALFF, and ReHo (Figure S3, Supporting Information). In contrast, genePC1 was positively correlated with GMV differences (*r*_s_ = 0.367, *p* = 4.36 × 10^−8^) (Figure 2C). The opposite relationships of whole gene expression with funcPC1 and GMV differences indicates that the distribution of gene expression in the cortex is differentially related to functional and structural difference patterns in MDD. This pattern of opposite relationships of gene expression with structural and functional brain abnormities was replicated using data from the UK Biobank (Figures S4 and S5, Supporting Information).

Because females are nearly twice as likely to develop depression than males, we explored whether there were sex‐related differences in the observed results. We therefore split the dataset into a male group (314 MDD patients and 327 healthy controls) and a female group (534 MDD patients and 437 healthy controls), and investigated the relationships of genePC1 with brain abnormalities in both groups separately. Although no significant structural or functional case‐control differences were observed in the two groups (presumably due to the decrease in statistical power), the distribution of interregional differences in the male or female group were similar to those identified in whole dataset (Figure S6, Supporting Information). Moreover, in both groups, gene expression showed opposite relationships with structural and functional differences, which were consistent with the results in the whole dataset (Figures S7–S9, Supporting Information). These results indicate that the pattern of opposite relationships did not show differences between males and females.

Moreover, age ranged from 18 to 65 years old in REST‐meta‐MDD dataset. To test age influences on the observed relationships, we divided the dataset into an older group (≥32 years old, 420 MDD patients, and 433 healthy controls) and younger group (<32 years old, 428 MDD patients, and 361 healthy controls) (Figure S10, Supporting Information), based on a median split. In both groups, the interregional structural and functional case‐control differences were highly positively associated with those identified in the whole dataset (Figure S11, Supporting Information). In the older group, we found opposite relationships of genePC1 with funcPC1 and GMV differences, which were consistent with the results in the whole dataset (Figure S12, Supporting Information). In contrast, genePC1 showed weak positive correlations with funcPC1 and GMV differences in the younger group (Figure S13, Supporting Information). These findings indicate that the relationships may tend to emerge in a middle‐aged population.

We then identified the transcriptional correlates of brain abnormalities in MDD. Expression on 210 cortical regions of each gene were related to the funcPC1 and GMV differences, respectively (Tables S1 and S2, Supporting Information). After Bonferroni multiple testing correction, gene expression of 412 genes showed significant positive correlations and 415 genes showed significant negative correlations with funcPC1 (funcPC1+ genes and funcPC1−gene respectively), (Figure 2D, Figure S14A, Supporting Information). It indicates that higher gene expression in cortical regions is associated with increased and decreased neuronal activity in MDD for funcPC1+ and funcPC1− genes, respectively. For GMV, we identified 338 positively and 298 negatively related genes, which we termed GMV+ genes and GMV− genes, respectively, indicating higher gene expression in cortical regions with increased and decreased GMV in MDD, respectively (Figure 2E, Figure S14B, Supporting Information).

### 2.3 Relationships of Transcriptional Correlates with Gene Dysregulation

**Figure 3:**
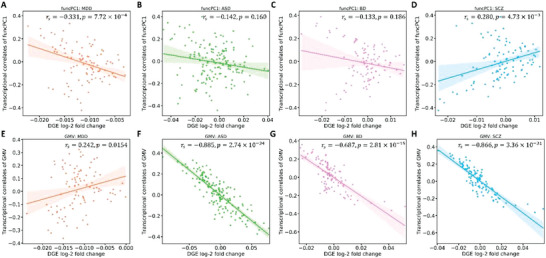
The correlations of the transcriptional correlates of brain abnormalities with differential gene expression (DGE) values for 100 gene bins. A–D) show the correlations for funcPC1. E–H) show the correlations for GMV. FuncPC1 is the first principal component of effect sizes for functional brain measures, and GMV indicates gray matter volume. Results are shown for major depressive disorder (MDD), autism spectrum disorder (ASD), bipolar disorder (BP), and schizophrenia (SCZ).

Following the approach of Gandal et al.,^[^ ^20^ ^]^ we obtained the meta‐analytic estimates of differential gene expression (DGE) in postmortem brain tissues of patients with major psychiatric disorders including MDD, schizophrenia (SCZ), autism spectrum disorder (ASD), and bipolar disorder (BP) (Table S3, Supporting Information). We first related in vivo transcriptional correlates of brain abnormalities to post‐mortem gene dysregulation in cortex of patients with MDD for genes. DGE values of genes for MDD were negatively correlated with their correlations with funcPC1, but not significantly correlated with transcriptional correlates of GMV differences (Figure S15, Supporting Information). However, DGE values of most genes were distributed around zero, which could cause bias for estimating correlations, so we applied bin‐based correlation analysis to increase the statistical power.^[^ ^17^ ^]^ When ≈10 000 genes were divided into 100 bins, the genes with similar transcriptional correlates of brain abnormalities were merged into a bin, which avoided the DGE values of too many genes gathering at a narrow range around zeros.^[^ ^17^ ^]^ We found a significant negative correlation with the transcriptional correlates of funcPC1, but a significant positive correlation with the transcriptional correlates of GMV differences (**Figure** **3**A,E). The finding indicates that the transcriptional correlates of in vivo MDD‐related brain abnormalities from healthy AHBA donors captured the information of differential gene expression in ex vivo brain tissue from MDD patients.

Through bin‐based correlation analysis, we further observed the DGE values of ASD and BD were not significantly associated with the transcriptional correlates of funcPC1 (Figure 3B,C), whereas the DGE values of SCZ showed a significant positive correlation with the transcriptional correlates of funcPC1 (Figure 3D), which was opposite to the finding by MDD in Figure 3A. In addition, the DGE values of ASD, BD, and SCZ showed strong negative correlations with the transcriptional correlates of MDD‐related GMV differences (Figure 3F–H), which were also in opposite direction with the finding by MDD in Figure 3E. This indicated that the transcriptional correlates of MDD‐related brain abnormalities can capture the gene dysregulation of other psychiatric disorders, and the direction of relationships for MDD were opposite to ASD, BD, and SCZ. Comparable results were obtained at other bin numbers: 50, 150, and 200 gene bins (Table S4, Supporting Information).

### 2.4 Gene Set Enrichment

**Figure 4:**
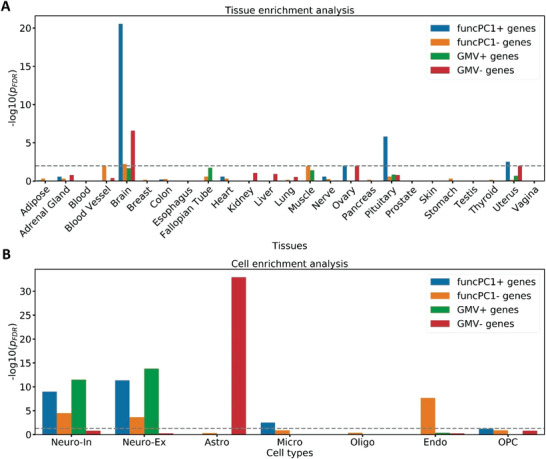
Enrichment analysis for tissues and cell types. A) Tissue specific expression analysis; B) Cell enrichment analysis. *p*_FDR_ is the adjusted *p* value after FDR multiple testing corrections. FuncPC1 is the first principal component of effect sizes for functional brain measures, and GMV indicates gray matter volume. Genes positively and negatively related to funcPC1 and GMV differences are defined as funcPC1+, funcPC1− genes, GMV+, and GMV− genes. Neuro‐In, inhibitory neuron; neuro‐Ex, excitatory neurons; Asto, astrocytes; endo, endothelial; mic, microglia; oligo, oligodendrocytes; OPC, oligodendrocyte precursor cells.

We first conducted the tissue‐specific expression analysis (TSEA).^[^ ^21^ ^]^ All of our identified genes were significantly enriched for brain tissue, especially funcPC1+ and GMV− genes (**Figure** **4**A). We also conducted the cell‐type expression analysis based on the study by Seidlitz et al.^[^ ^22^ ^]^ The GMV− genes were specifically enriched for astrocytes, whereas the other three gene sets were significantly enriched for inhibitory and excitatory neurons (Figure 4B). Moreover, the funcPC1+ and funcPC1− genes also showed significant enrichment for microglia and endothelial cells, respectively.

In addition, we selected up‐ and downregulated genes for psychiatric disorders with the largest 500 positive and negative DGE values. We tested whether genes transcriptionally related to funcPC1 or GMV differences were enriched for genes that were highly upregulated and downregulated in psychiatric disorders (Figure S16, Supporting Information). The funcPC1+, funcPC1−, and GMV− genes were enriched for MDD downregulated genes, but not for MDD upregulated genes. In addition, widespread significant enrichments were identified for genes that were up‐ and downregulated in ASD, BP, and SCZ. Of them, GMV− genes showed the strongest enrichment for upregulated genes for BP and SCZ. Comparable results were observed using other thresholds (the largest 300–800 positive and negative DGE values) to define up‐ and downregulated genes for psychiatric disorders (Figure S17, Supporting Information).

**Figure 5:**
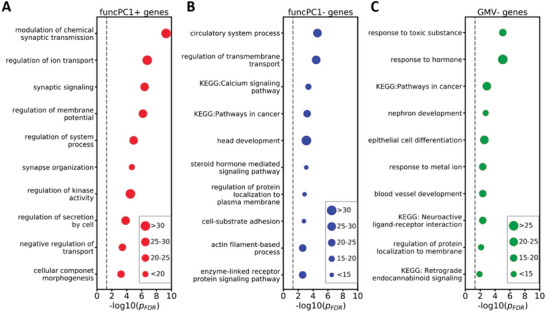
Top 10 enrichment pathways from the enrichment analysis for the gene ontology (GO) biological processes and Kyoto Encyclopedia of Genes and Genomes (KEGG) for different gene sets: A) Genes positively related to the first component of effect sizes for functional measures (funcPC1+ genes); B) Genes negatively related to the first component of effect sizes for functional measures (funcPC1‐ genes); C) Genes negatively related to GMV differences (GMV− genes). *p*_FDR_ is the adjusted *p* value after FDR multiple testing corrections. Node size is proportional to the number of input genes included in that term.

We also conducted enrichment analysis for biological pathways including gene ontology (GO) biological processes and Kyoto Encyclopedia of Genes and Genomes (KEGG) pathways. The top 10 significant enrichment pathways are presented in **Figure** **5**. For funcPC1+ genes, we observed enriched pathways such as synaptic transmission (i.e., modulation of chemical synaptic transmission, synaptic signaling, and synapse organization) and transport processes (i.e., regulation of ion transport, and negative regulation of transport) (Figure 5A). For funcPC1− genes, significant enrichment was mainly found for signaling pathways: Calcium signaling pathway, steroid hormone mediated signaling pathway, and enzyme‐linked receptor protein signaling pathway (Figure 5B). In contrast, GMV− genes showed different enrichment terms, such as response to metal ion, neuroactive ligand‐receptor interaction, and retrograde endocannabinoid signaling (Figure 5C), whereas GMV+ genes were only enriched for three pathways: inorganic ion transmembrane transport, sensory organ morphogenesis, and skeletal system development.

## 3 Conclusion

In this study, we compared structural and functional brain abnormalities between MDD cases and controls. We observed similar case‐control differences for ALFF, fALFF, and ReHo, and extracted the first principal component, funcPC1, which explained 71% variance of functional brain abnormalities in MDD, whereas smaller case‐control differences were observed for GMV. We then related gene expression to funcPC1 and GMV differences, and found that whole gene expression (genePC1) was negatively associated with funcPC1, but positively associated with GMV differences. These opposite relationships were replicated in the UK Biobank, indicating that they exist in Asian and European populations. Moreover, we identified genes positively and negatively related to funcPC1 as well as GMV differences: funcPC1+, funcPC1−, GMV+, and GMV− genes. The identified genes were enriched for the brain tissue, cortical cells, and genes that were downregulated in MDD. Furthermore, MDD‐related genes showed different enrichment for biological pathways between brain function and structure. Together, these findings provide evidence for distinct genetic contributions to functional and structural brain abnormalities in MDD.

We found many functional abnormalities in MDD that were largely in line with previous studies.^[^ ^6b,6c^ ^]^ The abnormal regions, such as the anterior cingulate cortex and prefrontal cortex, are implicated in emotion regulation and cognitive functions.^[^ ^23^ ^]^ However, we did not observe significant differences in GMV. Nevertheless, it showed the distribution of structural brain abnormalities in MDD across cortical regions, which was consistent with the previous ENIGMA meta‐analysis of structural brain abnormalities (Supporting Information).^[^ ^6e^ ^]^ When relating gene expression to case‐control differences in both the REST‐meta‐MDD and UK Biobank datasets, we found a negative correlation with brain function but a positive correlation with brain structure. This suggests that the gene expression patterns across the brain capture biologically meaningful and distinct information about MDD‐related structural and functional brain abnormalities. A recent study also observed a positive correlation between gene expression and differences of morphometric similarity networks.^[^ ^18^ ^]^ However, the study included a limited sample size (217 MDD patients and 208 healthy controls) and did not investigate brain function. Our results therefore extend these findings, and show that cortical gene expression can have divergent influences on structural versus functional brain abnormalities.^[^ ^24^ ^]^ Interestingly, this previous study found MDD participants exhibited decreased morphometric similarity in the left superior frontal cortex, and increased morphometric similarity in the left medial orbitofrontal cortex, isthmus cingulate cortex, and four occipital regions, which were not consistent with our identified GMV case‐control differences. These differences in results are likely the consequence of different definitions of brain structure. The morphometric similarity feature was created by combining seven features from the T1w and DWI images, including surface area, cortical thickness, gray matter volume, Gaussian curvature, mean curvature, fractional anisotropy, and mean diffusivity, while we only considered gray matter volume. Another previous study combined structural and functional correlates of depression and negative affect across three datasets, but they ignored the potential differences in transcriptional correlates of brain structure and function. Moreover, they conducted statistical analysis in depressed individuals based on lifetime presence of depression or healthy adults with negative affect measures, so their findings do not reflect disease status at the time of MRI scanning.^[^ ^17^ ^]^ Our results confirm that relations between gene expression and brain structure and function also occur during a depressive episode.

Through gene set enrichment analysis, we found that the identified genes showed specific expression in brain tissue and cortical cells, providing credibility for the observed transcriptome–neuroimaging relationships.^[^ ^13^ ^]^ Interestingly, except for the GMV− genes, all gene sets were enriched for inhibitory and excitatory neurons, which have previously been related to depression.^[^ ^25^ ^]^ For example, the somatostatin (SST) interneuron is one of the major inhibitory neuron types, which primarily target the apical dendrites of pyramidal neurons and provide synaptic and extrasynaptic inhibition.^[^ ^26^ ^]^ Reduced dendritic inhibition from SST interneurons plays key roles in treatment‐resistant depression.^[^ ^27^ ^]^ Moreover, the gene expression by SST interneurons was significantly lower in postmortem cortical tissue from depressed individuals.^[^ ^28^ ^]^ The parvalbumin (PV) neuron is another type of inhibitory neurons, which coordinates synchronous neuronal firing,^[^ ^29^ ^]^ and reductions in PV neurons in the prefrontal cortex were observed from postmortem MDD patients.^[^ ^30^ ^]^ In addition, excitatory neurons were shown to be involved in reward circuits in depression.^[^ ^31^ ^]^ For non‐neurons, GMV− genes were significantly enriched in astrocytes, which influence synapse formation and elimination,^[^ ^32^ ^]^ and have been linked to MDD.^[^ ^33^ ^]^

The identified genes were enriched for genes that were downregulated in MDD, and the transcriptional correlates of structural and functional brain abnormalities showed positive and negative correlations with gene dysregulation in postmortem tissue samples from MDD patients, respectively. This is in line with the identified relationships of whole gene expression with MDD‐related differences in the cortex. Interestingly, the transcriptional correlates of MDD‐related brain abnormities also showed significant associations with gene dysregulation in other psychiatric disorders. These findings could be in line with genetic pleiotropy, where a single gene influences multiple phenotypic traits,^[^ ^34^ ^]^ but could also be the result of a causal association, where certain abnormal gene expression in cortical regions could cause other psychiatric disorders, which may in turn cause depression.

The enrichment analysis was conducted for GO biological processes and KEGG pathways, and genes related to functional and structural abnormalities showed enriched biological pathways implicated in depression. For example, funcPC1+ genes were enriched for pathways related to synaptic transmission. Previous studies have proposed the hypothesis that the homeostatic mechanisms that control synaptic plasticity is disrupted in depression, resulting in destabilization and loss of synaptic connections in mood and emotion circuitry.^[^ ^35^ ^]^ The funcPC1− genes were enriched for signaling pathways, such as steroid hormone mediated signaling pathway. Previous research indicated that the depression may have a bidirectional association with altered steroid hormone secretion, leading to a vicious circle of more severe depression and more profound disturbances in steroid hormone signaling.^[^ ^36^ ^]^ For GMV− genes, we observed enriched KEGG pathways of neuroactive ligand‐receptor interaction and retrograde endocannabinoid signaling, which modulate various synaptic neurotransmissions or neural functions, including cognition, motor control, and pain^[^ ^37^ ^]^ and are related to MDD.^[^ ^38^ ^]^ Overall, genes related to functional and structural abnormalities showed widespread and distinct enriched biological pathways.

While our study used a larger sample size, and first reported the distinct transcriptome‐neuroimaging relationships between structural and functional abnormalities, there are also some limitations that need to be considered. First, AHBA expression data was measured based on tissues from six post‐mortem adults with no known neuropsychiatric or neuropathological history. It was therefore not possible to examine the changes of regional expression levels of genes in depressed patients. Moreover, the small sample size (six donors), sex unbalance (one female and five males), and only two donors providing samples in the left hemisphere may cause some bias in our statistical analysis. Second, only GMV was used to measure brain structure in the REST‐meta‐MDD dataset, which only measures one aspect of brain structure. Nevertheless, we replicated this in the UK Biobank, in which we also observed a positive correlation of whole gene expression with case‐control differences for cortical thickness. Third, although the relationships of whole gene expression with structural and functional abnormalities in MDD has been replicated in UK Biobank, the replication of transcriptional correlates for 15 633 individual genes was difficult to be implemented. This may be resulted by the biased definition of depressed individuals in UK Biobank dataset, which only considers two core symptoms of depression.

In summary, this study found opposite relationships of gene expression with structural and functional brain abnormalities in individuals with MDD. The transcriptional correlates of brain abnormalities showed significant correlations with gene dysregulation in postmortem tissue samples from MDD patients. Moreover, we identified genes involved in brain abnormalities, which were enriched for brain tissues, cortical, cells and biological pathways. These results highlight the importance of cortical gene expression for the development of brain abnormalities in MDD, and improve the understanding of underlying neural mechanisms.

## 4 Experimental Section

### Participants

In the main analysis, publicly available data with brain scans from the REST‐meta‐MDD project were used.^[^ ^39^ ^]^ The project totally included 1300 patients diagnosed with MDD and 1128 healthy controls. From them, 848 patients diagnosed with MDD and 794 healthy from 17 sites in China were extracted for group comparison analysis (Figure S18, Supporting Information). Age and sex did not have difference between the MDD and control group (age: *t* = −0.75, *p* = 0.94, sex: *χ* ^2^ = 2.8, *p* = 0.094). Individuals with MDD were diagnosed as having current depressive status by experienced psychiatric physicians based on ICD10 or DSM‐IV. All participants provided written informed consent and the project was approved by local Institutional Review Boards (for details see Table S5, Supporting Information).

The REST‐meta‐MDD dataset was comprised of East Asian individuals from China. To assess whether the results can be generalized to other ethnic populations, the analyses were replicated in a European‐ancestry sample from the UK Biobank (802 depressed individuals and 802 controls) (Figure S19, Table S6, Supporting Information), matched for age, sex, intelligence, and educational attainment. More information about the sample selection, and phenotypic measures in the UK Biobank sample can be found in Supporting Information.

### Imaging Procedures

Structural and functional MRI data were collected from 17 scanning sites using different 3T or 1.5T scanners, and different parameters were used for imaging acquisition (Table S5, Supporting Information). A MATLAB‐ and SPM‐based pipeline, DPARSF,^[^ ^40^ ^]^ was used to preprocess the resting‐state functional MRI (rsfMRI).^[^ ^39a^ ^]^ Main procedures included removing the first 10 time points, slice‐timing correction, realignment, regressing out head motion effects using the Friston 24 parameter model,^[^ ^41^ ^]^ and normalization from individual native space to MNI space using DARTEL tool.^[^ ^42^ ^]^ After realignment, individual T1 weighted images were co‐registered to the mean functional image and then segmented into gray matter (GM) and white matter. GM images were also modulated and spatially normalized by the DARTEL tool.

After preprocessing, three main functional brain measures (ALFF, fALFF, and ReHo) were calculated using rsfMRI data. Next, all functional images were Z standardized and then smoothed with a 3 mm full‐width at half maximum (FWHM) Gaussian kernel. Similarly, GM images were also smoothed with the 3 mm FWHM Gaussian kernel. Finally, smoothed GM and three functional images were mapped to the BN atlas to extract GMV, ALFF, fALFF, and ReHo of 210 cortical regions, respectively. More details about the imaging acquisition and processing can be found in the study by Yan et al.^[^ ^39a^ ^]^

The UK Biobank data were analyzed using a series of imaging processing steps which can be found in Supporting Information.

### Estimation of Regional Gene Expression

The AHBA microarray DNA data (http://human.brain‐map.org), comprising gene expression measures from a total of 3702 tissue samples by probes were used.^[^ ^11^ ^]^ The tissues were collected from six human donor brains 24 to 57 years of age with no known neuropsychiatric or neuropathological history (five males and one female). Of the donors, there were three Caucasian, two African‐American, and one Hispanic. Then, the abagen toolbox^[^ ^43^ ^]^ in Python was used to create a whole‐genome expression atlas by probe‐to‐gene and sample‐to‐region strategies using the following steps: 1) intensity‐based filtering of microarray probes to remove those that do not exceed background noise of 50%; 2) selection of a representative probe for each gene across each hemisphere; 3) matching of microarray samples to brain parcels from the BN atlas; and 4) normalization, and aggregation within parcels and across donors.^[^ ^12^ ^]^ This resulted in a 2D matrix that showed gene expression levels of 15 633 genes on 210 BN cortical regions. In addition, another cortical parcellation with 68 cortical regions, the DK atlas, was used to extract AHBA gene expression matrix for replication analysis.

### Statistical Analysis

Two‐sample *t*‐tests were used to obtain the case‐control differences in GMV, ALFF, fALFF, and ReHo based on the BN atlas. T‐scores were then converted to Cohen's d effect sizes for ease of interpretation. The covariates, including age, sex, education, and head motion, were regressed out of the brain measures. The FDR correction was applied to correct for multiple comparisons using the Benjamini and Hochberg method with a threshold of *p* = 0.05. For a given brain measure, Cohen's d across 210 cortical regions was used to quantify structural or functional abnormalities in MDD. PCA was used to extract the first PC of effect sizes for the three functional measures, defined as funcPC1 that explained most variance of functional abnormalities. The first PC was also extracted from the gene expression matrix, defined as genePC1.

To test the relationship of whole gene expression of 15 633 genes for 210 cortical regions with the corresponding brain abnormalities, the correlation of genePC1 with GMV differences and funcPC1 was examined using the Spearman's rank correlation analysis.

To replicate the whole transcriptome‐neuroimaging relationships, identical analyses were conducted in the UK Biobank dataset (Supporting Information). In addition, possible effects of sex and age were tested on the transcriptome‐neuroimaging relationships (Supporting Information). Moreover, relationships between cortical expression distribution of individual genes and brain abnormalities were examined, indicating individual gene's transcriptional correlates of MDD‐related brain abnormalities. A Bonferroni multiple comparison correction was applied to select the genes significantly positively or negatively related to GMV differences, defined as GMV+ genes or GMV− genes. Similarly, genes related to funcPC1 were defined as funcPC1+ or funcPC1‐ genes.

### Differential Gene Expression Analysis in Major Psychiatric Disorders

Following the approach of Gandal et al.,^[^ ^20^ ^]^ the DGE estimates in postmortem brain tissues of patients with major psychiatric disorders were obtained. In short, through linear mixed effects modeling, standardized beta coefficients were calculated as DGE values, which quantified the degree that a gene is up‐ or downregulated for a given psychiatric population. The tissues were sampled mainly from the prefrontal and anterior cingulate cortex of MDD patients, and the prefrontal cortex only for other psychiatric patients.

The ≈10 000 overlapping genes between AHBA and Gandal et al.^[^ ^20^ ^]^ datasets were obtained, and examined the associations between transcriptional correlates of brain abnormities in MDD and DGE values for psychiatric disorders for these common genes using Spearman's rank correlation analysis. It had been suggested that linking transcriptional correlates to DGE values directly for all genes could cause the bias for estimating correlation because of the noise,^[^ ^17^ ^]^ the bin‐based correlation analysis (Supporting Information) was further applied. In short, the common genes were ranked by their correlations with brain abnormalities (transcriptional correlates) in descending order. The ranked genes were then divided into 100 bins, which merged the genes with similar transcriptional correlates of brain abnormalities into a bin. For example, the genes with highest positive and negative correlations were divided into the first and 100th gene bin, respectively. The average transcriptional correlates and DGE values were calculated for each bin. For 100 gene bins, the relationships of average transcriptional correlates of in vivo brain abnormalities were examined with ex vivo DGE values for psychiatric disorders. Alternative numbers of bins (*N* = 50, 150, and 200) were also applied to test the stability across choices of bin numbers.

### Enrichment Analysis

Gene set enrichment analysis was applied to identify functional annotations of the identified genes related to brain abnormalities in MDD (GMV+ genes, GMV− genes, funcPC1+ genes, and funcPC1− genes). In TSEA,^[^ ^21^ ^]^ a specificity index probability (*p*_SI_ = 0.05, permutation corrected) and Fisher's exact test with FDR‐BH correction were used to determine how likely our identified genes were to be specifically expressed in a given tissue. Next, cell‐type expression analysis were conducted based on the study by Seidlitz et al.^[^ ^22^ ^]^ They provided gene sets related to seven canonical cortical cell classes: inhibitory neurons, excitatory neurons, astrocytes, microglia, endothelial cells, oligodendrocyte precursors, and oligodendrocytes (Table S7, Supporting Information). The GSEApy package^[^ ^44^ ^]^ was used in Python to identify the cell types that genes related to brain abnormalities were enriched for. Moreover, the genes with the largest 300–800 positive and negative DGE values were selected as up‐ and downregulated gene sets for psychiatric disorders. Using the enrichment analysis, this work tested whether up‐ and downregulated genes for psychiatric disorders were expressed most in cortical regions that showed structural and functional abnormalities in MDD. In addition, the Enrichr tool available online (http://amp.pharm.mssm.edu/Enrichr) was used to identify enrichment pathways for the gene ontology (GO) biological processes and Kyoto Encyclopedia of Genes and Genomes (KEGG) pathways with FDR‐BH correction.^[^ ^44^ ^]^

## Conflict of Interest

The authors declare no conflict of interest.

## Supporting information

Supporting Information

Supplemental Table

## Data Availability

The data that support the findings of this study are available from UK Biobank. Restrictions apply to the availability of these data, which were used under license for this study. Data are available at https://www.ukbiobank.ac.uk/ with the permission of UK Biobank.

## References

[1] R. C. Kessler , P. Berglund , O. Demler , R. Jin , D. Koretz , K. R. Merikangas , A. J. Rush , E. E. Walters , P. S. Wang , JAMA, J. Am. Med. Assoc. 2013, 289, 3095. 10.1001/jama.289.23.3095 12813115

[2] A. F. Awedew , H. Han , B. Abbasi , M. Abbasi‐Kangevari , M. B. Ahmed , O. Almidani , E. Amini , J. Arabloo , A. M. Argaw , S. S. Athari , D. Atlaw , M. Banach , A. Barrow , A. S. Bhagavathula , V. S. Bhojaraja , B. Bikbov , B. B. A. Bodicha , N. S. Butt , F. L. C. dos Santos , O. Dadras , X. Dai , L. P. Doan , S. Eftekharzadeh , A. Fatehizadeh , T. Garg , T. G. Gebremeskel , M. E. Getachew , S.‐H. Ghamari , S. A. Gilani , M. Golechha , et al., Lancet Healthy Longevity 2022, 3, e754. 36273485 10.1016/S2666-7568(22)00213-6 PMC9640930

[3] P. F. Sullivan , M. C. Neale , K. S. Kendler , Am. J. Psychiatry 2000, 157, 1552. 11007705 10.1176/appi.ajp.157.10.1552

[4] D. M. Howard , M. J. Adams , T. K. Clarke , J. D. Hafferty , J. Gibson , M. Shirali , J. R. I. Coleman , S. P. Hagenaars , J. Ward , E. M. Wigmore , C. Alloza , X. Shen , M. C. Barbu , E. Y. Xu , H. C. Whalley , R. E. Marioni , D. J. Porteous , G. Davies , I. J. Deary , G. Hemani , K. Berger , H. Teismann , R. Rawal , V. Arolt , B. T. Baune , U. Dannlowski , K. Domschke , C. Tian , D. A. Hinds , M. Trzaskowski , et al., Nat. Neurosci. 2019, 22, 343. 30718901 10.1038/s41593-018-0326-7 PMC6522363

[5] D. F. Levinson , S. Mostafavi , Y. Milaneschi , M. Rivera , S. Ripke , N. R. Wray , P. F. Sullivan , Biol. Psychiatry 2014, 76, 510. 25201436 10.1016/j.biopsych.2014.07.029 PMC4740915

[6] a) S. M. Grieve , M. S. Korgaonkar , S. H. Koslow , E. Gordon , L. M. Williams , Neuroimage Clin. 2013, 3, 332; 24273717 10.1016/j.nicl.2013.08.016 PMC3814952

[7] C. Scharinger , U. Rabl , L. Pezawas , S. Kasper , World J. Biol. Psychiatry 2011, 12, 474. 21830992 10.3109/15622975.2011.596220

[8] S. Schmitt , T. Meller , F. Stein , K. Brosch , K. Ringwald , J. K. Pfarr , C. Bordin , N. Peusch , O. Steinsträter , D. Grotegerd , K. Dohm , S. Meinert , K. Förster , R. Redlich , N. Opel , T. Hahn , A. Jansen , A. J. Forstner , F. Streit , S. H. Witt , M. Rietschel , B. Müller‐Myhsok , M. M. Nöthen , U. Dannlowski , A. Krug , T. Kircher , I. Nenadić , Psychol. Med 2021, 1. https://pubmed.ncbi.nlm.nih.gov/33827729/ 10.1017/S0033291721001082 PMC9811276 33827729

[9] D. Yüksel , B. Dietsche , A. J. Forstner , S. H. Witt , R. Maier , M. Rietschel , C. Konrad , M. M. Nöthen , U. Dannlowski , B. T. Baune , T. Kircher , Prog. Neuro‐Psychopharmacol. Biol. Psychiatry 2017, 79, 67. 10.1016/j.pnpbp.2017.06.010 28624581

[10] O. Y. Naumova , M. Lee , S. Y. Rychkov , N. V. Vlasova , E. L. Grigorenko , Child Dev. 2013, 84, 76. 23145569 10.1111/cdev.12014 PMC3557706

[11] M. J. Hawrylycz , E. S. Lein , A. L. Guillozet‐Bongaarts , E. H. Shen , L. Ng , J. A. Miller , L. N. Van De Lagemaat , K. A. Smith , A. Ebbert , Z. L. Riley , C. Abajian , C. F. Beckmann , A. Bernard , D. Bertagnolli , A. F. Boe , P. M. Cartagena , M. Mallar Chakravarty , M. Chapin , J. Chong , R. A. Dalley , B. D. Daly , C. Dang , S. Datta , N. Dee , T. A. Dolbeare , V. Faber , D. Feng , D. R. Fowler , J. Goldy , B. W. Gregor , et al., Nature 2012, 489, 391. 22996553 10.1038/nature11405 PMC4243026

[12] A. Arnatkevic̆iūtė , B. D. Fulcher , A. Fornito , NeuroImage 2019, 189, 353. 30648605 10.1016/j.neuroimage.2019.01.011

[13] J. Fu , F. Liu , W. Qin , Q. Xu , C. Yu , Cereb. Cortex 2020, 30, 3655. 32186704 10.1093/cercor/bhz333

[14] J. Ritchie , S. P. Pantazatos , L. French , NeuroImage 2018, 174, 504. 29567503 10.1016/j.neuroimage.2018.03.027 PMC6450807

[15] a) J. Richiardi , A. Altmann , A. C. Milazzo , C. Chang , M. M. Chakravarty , T. Banaschewski , G. J. Barker , A. L. W. Bokde , U. Bromberg , C. Büchel , P. Conrod , M. Fauth‐Bühler , H. Flor , V. Frouin , J. Gallinat , H. Garavan , P. Gowland , A. Heinz , H. Lemaître , K. F. Mann , J. L. Martinot , F. Nees , T. Paus , Z. Pausova , M. Rietschel , T. W. Robbins , M. N. Smolka , R. Spanagel , A. Ströhle , G. Schumann , et al., Science 2015, 348, 1241; 26068849 10.1126/science.1255905 PMC4829082

[16] a) R. Romero‐Garcia , V. Warrier , E. T. Bullmore , S. Baron‐Cohen , R. A. I. Bethlehem , Mol. Psychiatry 2019, 24, 1053; 29483624 10.1038/s41380-018-0023-7 PMC6755982

[17] K. M. Anderson , M. A. Collins , R. Kong , K. Fang , J. Li , T. He , A. M. Chekroud , B. T. Thomas Yeo , A. J. Holmes , Proc. Natl. Acad. Sci. U. S. A. 2020, 117, 25138. 32958675 10.1073/pnas.2008004117 PMC7547155

[18] J. Li , J. Seidlitz , J. Suckling , F. Fan , G. J. Ji , Y. Meng , S. Yang , K. Wang , J. Qiu , H. Chen , W. Liao , Nat. Commun. 2021, 12, 1647. 33712584 10.1038/s41467-021-21943-5 PMC7955076

[19] L. Fan , H. Li , J. Zhuo , Y. Zhang , J. Wang , L. Chen , Z. Yang , C. Chu , S. Xie , A. R. Laird , P. T. Fox , S. B. Eickhoff , C. Yu , T. Jiang , Cereb. Cortex 2016, 26, 3508. 27230218 10.1093/cercor/bhw157 PMC4961028

[20] M. J. Gandal , J. R. Haney , N. N. Parikshak , V. Leppa , G. Ramaswami , C. Hartl , A. J. Schork , V. Appadurai , A. Buil , T. M. Werge , C. Liu , K. P. White , S. Horvath , D. H. Geschwind , Science 2018, 359, 693. 32015716 10.1176/appi.focus.17103 PMC6996074

[21] J. D. Dougherty , E. F. Schmidt , M. Nakajima , N. Heintz , Nucleic Acids Res. 2010, 38, 4218. 20308160 10.1093/nar/gkq130 PMC2910036

[22] J. Seidlitz , A. Nadig , S. Liu , R. A. I. Bethlehem , P. E. Vértes , S. E. Morgan , F. Váša , R. Romero‐Garcia , F. M. Lalonde , L. S. Clasen , J. D. Blumenthal , C. Paquola , B. Bernhardt , K. Wagstyl , D. Polioudakis , L. de la Torre‐Ubieta , D. H. Geschwind , J. C. Han , N. R. Lee , D. G. Murphy , E. T. Bullmore , A. Raznahan , Nat. Commun. 2020, 11, 3358. 32620757 10.1038/s41467-020-17051-5 PMC7335069

[23] V. Gazzola , M. L. Spezio , J. A. Etzel , F. Castelli , R. Adolphs , C. Keysers , Proc. Natl. Acad. Sci. U. S. A. 2012, 109, E1657. 22665808 10.1073/pnas.1113211109 PMC3382530

[24] D. S. Scheepens , J. A. van Waarde , A. Lok , G. de Vries , D. A. J. P. Denys , G. A. van Wingen , Front. Psychiatry 2020, 11, 485. 32581868 10.3389/fpsyt.2020.00485 PMC7283615

[25] J. Persson , A. Wall , J. Weis , M. Gingnell , G. Antoni , M. Lubberink , R. Bodén , Psychiatry Res. Neuroimaging 2021, 315, 111327. 34246046 10.1016/j.pscychresns.2021.111327

[26] R. Tremblay , S. Lee , B. Rudy , Neuron 2016, 91, 260. 27477017 10.1016/j.neuron.2016.06.033 PMC4980915

[27] H. K. Yao , A. Guet‐McCreight , F. Mazza , H. Moradi Chameh , T. D. Prevot , J. D. Griffiths , S. J. Tripathy , T. A. Valiante , E. Sibille , E. Hay , Cell Rep. 2022, 38, 110232. 35021088 10.1016/j.celrep.2021.110232

[28] M. L. Seney , A. Tripp , S. McCune , D. A. Lewis , E. Sibille , Neurobiol. Dis. 2015, 73, 213. 25315685 10.1016/j.nbd.2014.10.005 PMC4394026

[29] G. Perlman , A. Tanti , N. Mechawar , Neurobiol. Stress 2021, 15, 100380. 34557569 10.1016/j.ynstr.2021.100380 PMC8446799

[30] G. Rajkowska , G. O'Dwyer , Z. Teleki , C. A. Stockmeier , J. J. Miguel‐Hidalgo , Neuropsychopharmacology 2007, 32, 471. 17063153 10.1038/sj.npp.1301234 PMC2771699

[31] S. M. Thompson , A. J. Kallarackal , M. D. Kvarta , A. M. Van Dyke , T. A. LeGates , X. Cai , Trends Neurosci. 2015, 38, 279. 25887240 10.1016/j.tins.2015.03.003 PMC4417609

[32] W. S. Chung , N. J. Allen , C. Eroglu , Cold Spring Harb. Perspect. Biol. 2015, 7, a020370. 25663667 10.1101/cshperspect.a020370 PMC4527946

[33] a) X. Zhou , Q. Xiao , L. Xie , F. Yang , L. Wang , J. Tu , Front. Mol. Neurosci. 2019, 12, 136; 31231189 10.3389/fnmol.2019.00136 PMC6560156

[34] J. Gratten , P. M. Visscher , Genome Med. 2016, 8, 78. 27435222 10.1186/s13073-016-0332-x PMC4952057

[35] a) R. S. Duman , G. K. Aghajanian , Science 2012, 338, 68. 23042884 10.1126/science.1222939 PMC4424898

[36] J. Nowacki , K. Wingenfeld , M. Kaczmarczyk , W. R. Chae , P. Salchow , I. Abu‐Tir , D. Piber , J. Hellmann‐Regen , C. Otte , Transl. Psychiatry 2020, 10, 109. 32313032 10.1038/s41398-020-0789-7 PMC7171120

[37] a) P. E. Castillo , T. J. Younts , A. E. Chávez , Y. Hashimotodani , Neuron 2012, 76, 70; 23040807 10.1016/j.neuron.2012.09.020 PMC3517813

[38] R. Mechoulam , L. A. Parker , Annu. Rev. Psychol. 2013, 64, 21. 22804774 10.1146/annurev-psych-113011-143739

[39] a) C. G. Yan , X. Chen , L. Li , F. X. Castellanos , T. J. Bai , Q. J. Bo , J. Cao , G. M. Chen , N. X. Chen , W. Chen , C. Cheng , Y. Q. Cheng , X. L. Cui , J. Duan , Y. R. Fang , Q. Y. Gong , W. Bin Guo , Z. H. Hou , L. Hu , L. Kuang , F. Li , K. M. Li , T. Li , Y. S. Liu , Z. N. Liu , Y. C. Long , Q. H. Luo , H. Q. Meng , D. H. Peng , H. T. Qiu , et al., Proc. Natl. Acad. Sci. U. S. A. 2019, 116, 9078; 30979801 10.1073/pnas.1900390116 PMC6500168

[40] Y. Chao‐Gan , Z. Yu‐Feng , Front. Syst. Neurosci. 2010, 4, 13. 20577591 10.3389/fnsys.2010.00013 PMC2889691

[41] K. J. Friston , S. Williams , R. Howard , R. S. J. Frackowiak , R. Turner , Magn. Reson. Med. 1996, 35, 346. 8699946 10.1002/mrm.1910350312

[42] J. Ashburner , NeuroImage 2007, 38, 95. 17761438 10.1016/j.neuroimage.2007.07.007

[43] R. D. Markello , A. Arnatkevičiūtė , J. B. Poline , B. D. Fulcher , A. Fornito , B. Misic , eLife 2021, 10, e72129. 34783653 10.7554/eLife.72129 PMC8660024

[44] M. V. Kuleshov , M. R. Jones , A. D. Rouillard , N. F. Fernandez , Q. Duan , Z. Wang , S. Koplev , S. L. Jenkins , K. M. Jagodnik , A. Lachmann , M. G. McDermott , C. D. Monteiro , G. W. Gundersen , A. Maayan , Nucleic Acids Res. 2016, 44, W90. 27141961 10.1093/nar/gkw377 PMC4987924

