# Supplementary material for: Gene Expression has Distinct Associations with Brain Structure and Function in Major Depressive Disorder
Source: Adv Sci (Weinh). 2023 Jan 13;10(7):2205486. doi: 10.1002/advs.202205486 (PMC9982587; doi:10.1002/advs.202205486)
Supplement: Supplementary file 1 — Supporting Information [file ADVS-10-2205486-s001.pdf]

ADVANCED SCIENCE
Open Access

# Supporting Information

for *Adv. Sci.*, DOI 10.1002/advs.202205486

Gene Expression has Distinct Associations with Brain Structure and Function in Major Depressive Disorder

*Shu Liu*, Abdel Abdellaoui, Karin J. H. Verweij and Guido A. van Wingen**

## Supporting Information (SI)

# Gene expression has distinct associations with brain structure and function in major depressive disorder

*Shu Liu*, the DIRECT Consortium, Abdel Abdellaoui, Karin J.H. Verweij, Guido A. van Wingen**

S. Liu, Dr. A. Abdellaoui, Prof. K. J.H. Verweij, Prof. G. A. van Wingen
Amsterdam UMC location, University of Amsterdam, Department of Psychiatry
Amsterdam Neuroscience, Amsterdam
Meibergdreef 5, 1100 DD Amsterdam, The Netherlands.

# Contents

**SI Methods** .......... 4

**Replication dataset (UK Biobank)** .......... 4

**Imaging procedures in UK Biobank** .......... 4

**Statistical analysis in UK Biobank** .......... 5

**Statistical analysis in sex differences** .......... 5

**Statistical analysis in age stratification** .......... 5

**Bin-based correlation analysis** .......... 6

**SI Results** .......... 7

**The robustness of GMV case-control differences** .......... 7

**The effects of GMV on case-control functional brain differences** .......... 7

**SI Figures** .......... 8

**Figure S1**. Case-control functional brain differences based on the Brainnetome (BN) atlas. .......... 8

**Figure S2**. Case-control structural and functional brain differences based on the Desikan-Killiany (DK) atlas. .......... 9

**Figure S3**. Relationships of gene expression with effect sizes for functional measures. .......... 10

**Figure S4.** Case-control structural and functional brain differences in major depressive disorder (MDD) based on UK Biobank. .......... 11

**Figure S5**. Relations of gene expression with case-control differences in UK Biobank. .......... 12

**Figure S6**. The relationships of structural and functional brain abnormalities observed in the male or female group with the abnormalities observed in whole dataset. .......... 13

**Figure S7**. Relations of gene expression with case-control differences in the male group. .......... 14

**Figure S8.** Relations of gene expression with three functional brain differences in the male group. .......... 15

**Figure S9.** Relations of gene expression with case-control differences in the female group. .......... 16

**Figure S10.** The distribution of age. .......... 17

**Figure S11**. The relationships of structural and functional brain abnormalities observed in the old or young group with the abnormalities observed in whole dataset. .......... 18

**Figure S12.** Relations of gene expression with case-control differences in the old group. .......... 19

**Figure S13**. Relations of gene expression with case-control differences in the young group. .......... 20

**Figure S14**. Manhattan plots showing associations between gene expression and cortical differences. .......... 21

**Figure S15.** The correlations of the transcriptional correlates of brain abnormities with differential gene expression (DGE) values for major depressive disorder (MDD) when all genes are individually included. .......... 22

**Figure S16.** Enrichment analysis for 500 up- and down-regulated genes for psychiatric disorders. 23

**Figure S17**. Enrichment analysis for up- and down-regulated genes at different thresholds (300-800) based on differential gene expression (DGE) analysis in psychiatric disorders. .......... 24

**Figure S18**. Sample selection of REST-meta-MDD dataset copied from the study by Yan et al. ... 25
**Figure S19**. Sample selection of UK Biobank dataset. ... 26
**Figure S20.** The flow chart of bin-based correlation analysis. ... 27
**Figure S21**. The relationship of structural brain abnormalities between REST-meta-MDD and ENIGMA dataset. ... 28
**Figure S22.** The effects of gray matter volume (GMV) on case-control functional brain differences. ... 29
**References** ... 30

## SI Methods

### Replication dataset (UK Biobank)

We used UK Biobank data with brain scans as replication dataset.[1] All participants provided written informed consent and the project was granted ethical approval (reference: 11/NW/0382) by the National Health Service North West Centre for Research Ethics Committee. As shown in **Figure S19**, a total of 802 individuals were identified as depressed individuals using the following criteria: (1) Participants reported the presence of one of the core symptoms of depression as *frequency of depressed mood* (Data Field: 2050) or *unenthusiasm / disinterest* (Data Field: 2060) *in the last 2 weeks prior to brain scanning* that has been present “more than half the days” or “nearly every day”, and (2) Data for these participants were available for the following variables: T1 and resting-state functional MRI data as well as covariates including fluid intelligence score (Data Field: 20016), educational attainment (Data Field: 6138), sex (Data Field: 31), and age when attended assessment centre (Data Field: 21003). It should be noted that the two items about depression mentioned above were the only two symptoms of depression available in the UK Biobank that reflect the mood state during the MRI assessment,[2] whereas a total of five out of nine symptoms are required to get a full diagnosis of major depressive disorder (MDD). The controls had MRI data and covariates available, but reported no presence (“not at all”) of negative mood symptom or a loss of disinterest in the last 2 weeks prior to brain scanning. Moreover, the controls did not have a history of MDD, and never diagnosed to have any mental health problems. Of this control group we randomly selected 802 individuals matching age, sex, intelligence, and educational attainment with those of the depressed individuals (**Table S6**).

### Imaging procedures in UK Biobank

As replication dataset, we used T1-weighted MRI and rsfMRI data from UK Biobank. The brain imaging scanners used were standard Siemens Skyra 3T running VD13A SP4 with a 32-channel head coil. T1 scanning lasted about 5 minutes with the following parameters: repetition time = 2000 ms; echo time = 2.1 ms; flip angle = 8°; matrix size = 256 × 256 mm; voxel size = 1 × 1 × 1 mm; number of slices = 208. The acquisition parameters for the rsfMRI data were TR = 735 ms, TE = 39 ms, flip angle = 52°, matrix size =88 × 88 mm, voxel size = 2.4 × 2.4 × 2.4 mm, number of slices = 64, and volumes = 490. A series of preprocessing procedures were applied for T1 and rsfMRI data (http://biobank.ctsu.ox.ac.uk/crystal/crystal/docs/brain_mri.pdf). UK Biobank provided cortical surface area (CSA) and cortical thickness (CT) of 66 regions based on the Desikan-Killiany (DK) atlas.[3] For rsfMRI data, we used the BRANT toolbox[4] to estimate the amplitude of low-frequency fluctuation (ALFF) and regional homogeneity (ReHo) to detect the regional intensity of spontaneous fluctuations in the BOLD signal, and

extracted region values based on the DK atlas. High-pass temporal filtering has been applied to the rsfMRI data in UK Biobank which can cause calculation bias for fractional ALFF (fALFF), so we did not include fALFF in statistical analysis.

### Statistical analysis in UK Biobank

We first used two-sample *t* tests to compare the neuroimaging measures for cortical thickness (CT), ALFF, and ReHo based on the DK atlas between depressed individuals and controls. The t-values were then converted to Cohen's d effect sizes for ease of interpretation. The covariates, including age, sex, education, intelligence, and head motion, were regressed out of the brain measures. Principle component analysis (PCA) was used to extract the first components of effect sizes for ALFF and ReHo (funcPC1). Moreover, AHBA transcriptome gene expression data were mapped to the DK atlas, and the first component pf gene expression matrix (genePC1) was also extracted. To identify the global transcriptome-neuroimaging relationships, we related the genePC1 to funcPC1 and CT differences, respectively.

### Statistical analysis in sex differences

We split the whole REST-meta-MDD dataset into the male group and female group. The male group consisted of 314 MDD patients (age range: 18 - 65 years old) and 327 healthy controls (age range: 18 – 64 years old). The female group was comprised of 534 MDD patients (age range: 18 - 65 years old) and 437 healthy controls (age range: 18 - 63 years old). The covariates, including age, education, and head motion, were regressed out of the brain measures. We then used two-sample *t* test to examine the structural and functional case-control differences in the male and female group, respectively, and associated case-control differences with genePC1.

### Statistical analysis in age stratification

We split the whole REST-meta-MDD dataset into an older group and younger group respective to the age of 32 years old. The younger group was comprised of 428 MDD patients and 361 healthy controls. The older group consisted of 420 MDD patients and 433 healthy controls (**Figure S10**). The covariates, including age, sex, education, and head motion, were regressed out of the brain measures. We then used two-sample t test to examine the structural and functional brain case-control differences in the older and younger group, respectively, and associated case-control differences with genePC1

## Bin-based correlation analysis

As shown in **Figure S20**, we conducted the bin-based correlation analysis to link transcriptional correlates of brain abnormalities to differential gene expression (DGE) values. For all AHBA genes, we obtained their relationships of interregional expression levels with brain abnormalities (**Figure S20A**). According to the study by Gandal et al., we also obtained the differential gene expression (DGE) values for psychiatric disorders. We overlapped the AHBA genes and DGE genes (**Figure S20B**). The overlapping genes (N) were then sorted by their correlations between gene expression and brain abnormalities. These ordered genes were then clustered into 100 bins (bin size n = N/100). In detail, the top n genes were included as the first bin, the second n genes were grouped as the second bin, and so on. For all n genes in a bin, we calculated average DGE values, as well as average correlations between gene expression and abnormalities, and associated them using Spearman's correlation analysis (**Figure S20C**).

# SI Results

## The robustness of GMV case-control differences

We obtained the CT differences between MDD patients and controls across cortical regions based on meta-analysis in ENIGMA dataset[5] and associated them with our identified GMV case-control differences. A significant positive correlation was observed between structural brain abnormalities from two studies (**Figure S21**). The consistency demonstrated that GMV case-control differences were robust and represented the distribution of structural brain abnormalities, although no significant differences were observed.

## The effects of GMV on case-control functional brain differences

While testing the case-control functional brain differences, the covariates, including age, sex, education, and head motion were regressed out of functional brain measures. However, changes in GMV may artificially inflate or mask changes in functional measures in the same brain region. To test the effects of GMV, we corrected the functional brain measures for total GMV (TGMV). Compared to original case-control differences without correcting for TGMV, the effect sizes for TGMV-corrected functional brain measures hardly changed (**Figure S22A-C**). In addition, for functional brain measures of each region, we further corrected them for their regional GMV, and still found small changes on effect sizes (**Figure S22D-F**). These results indicate that small changes in functional measures caused by GMV changes do not influence the case-control brain differences.

## SI Figures

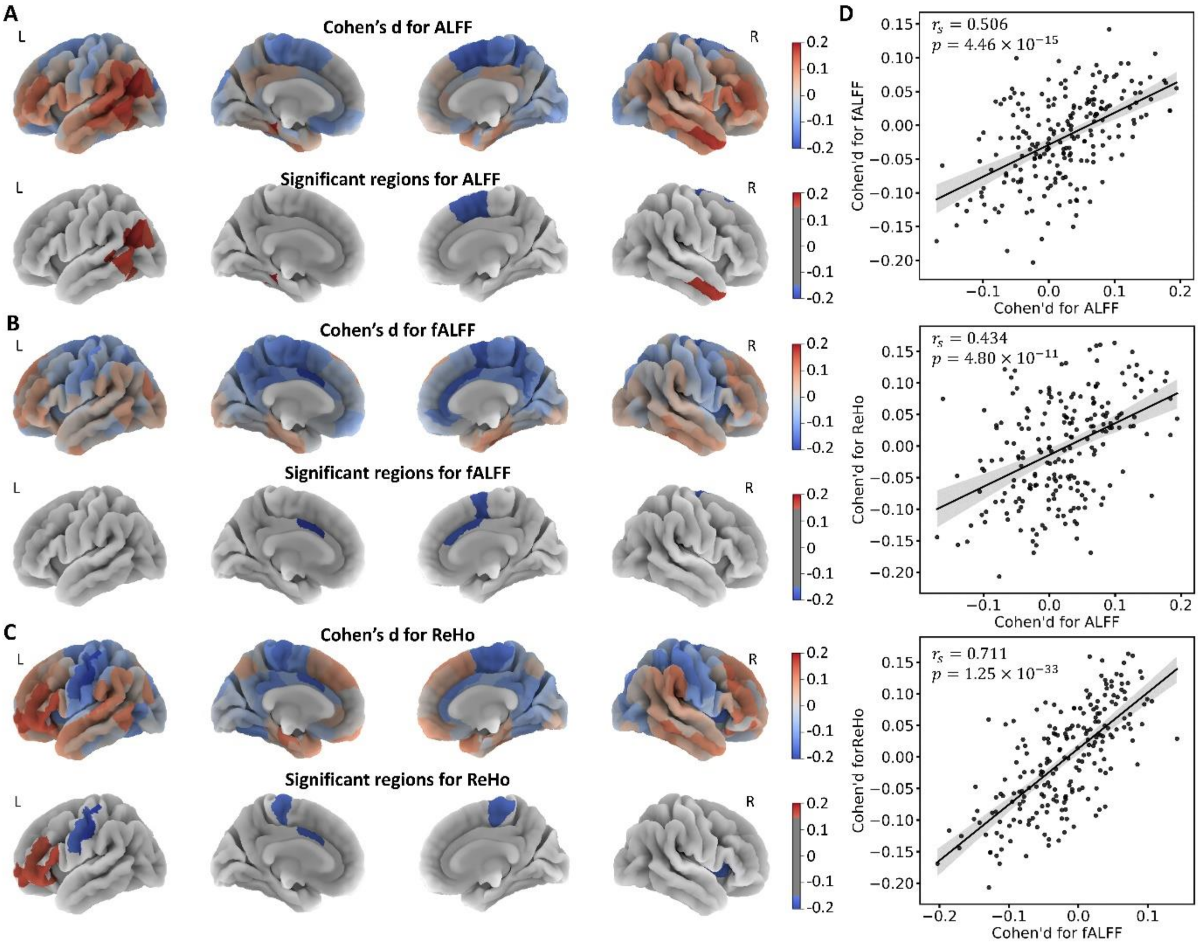


**Figure S1**. Case-control functional brain differences based on the Brainnetome (BN) atlas. **(A)** Differences (Cohen's d) for amplitude of low-frequency fluctuation (ALFF), and statistically significant regions; **(B)** Differences (Cohen's d) for fractional ALFF (fALFF), and statistically significant regions; **(C)** Differences (Cohen's d) for regional homogeneity (ReHo) and statistically significant regions; **(D)** the correlations between effect sizes for functional brain measures.

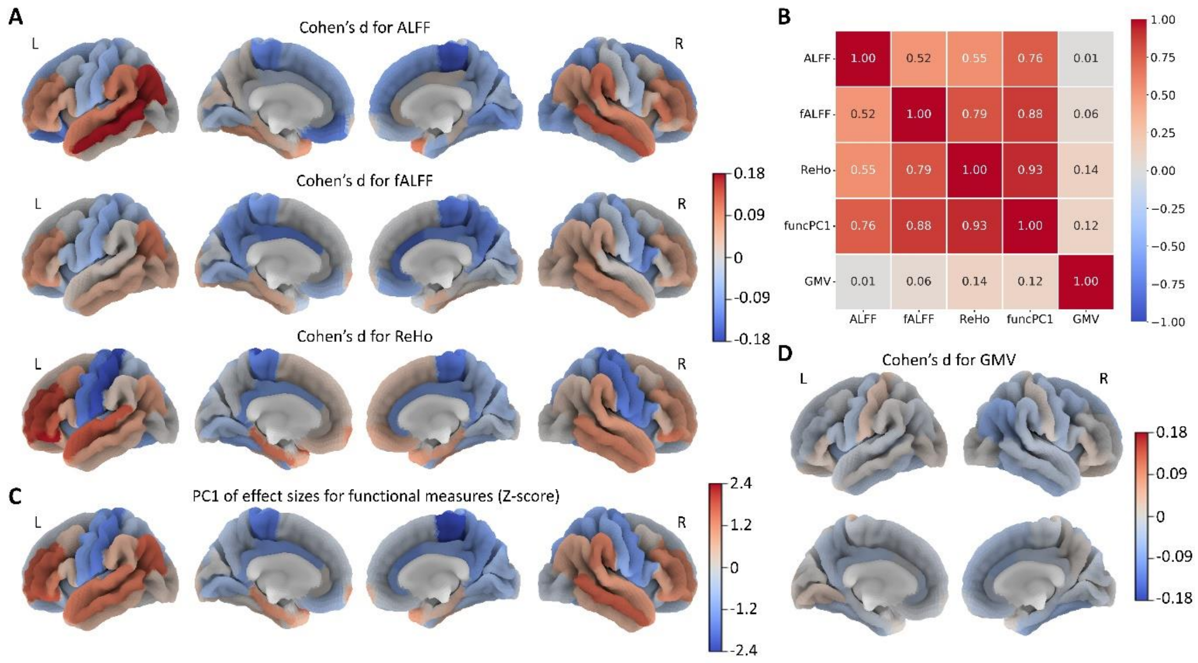


**Figure S2**. Case-control structural and functional brain differences based on the Desikan-Killiany (DK) atlas. **(A)** Differences (Cohen's d) for functional brain measures including amplitude of low-frequency fluctuation (ALFF), fractional ALFF (fALFF), and regional homogeneity (ReHo); **(B)** The correlations between case-control differences**;** **(C)** The first principle component of effect sizes for three functional measures (funcPC1) (Z-score normalization); **(D)** Differences (Cohen's d) for gray matter volume (GMV).

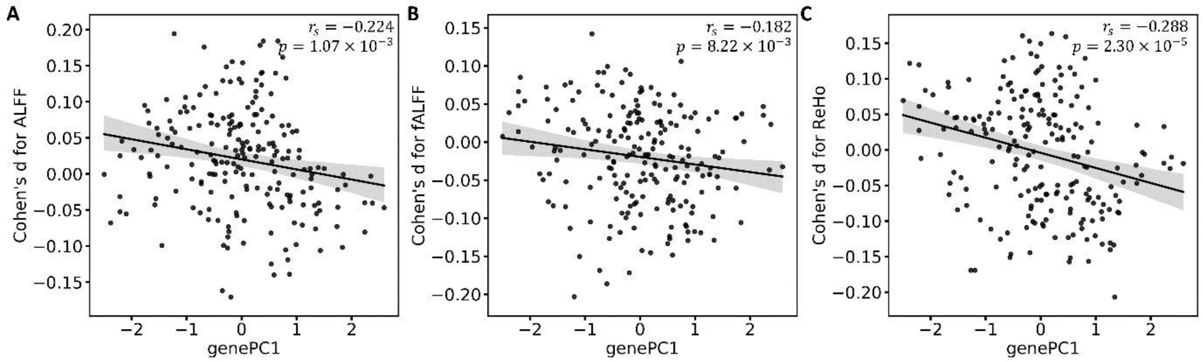


**Figure S3**. Relationships of gene expression with effect sizes for functional measures. **(A)** Amplitude of low-frequency fluctuation (ALFF); **(B)** Fractional ALFF (fALFF); **(C)** Regional homogeneity (ReHo).

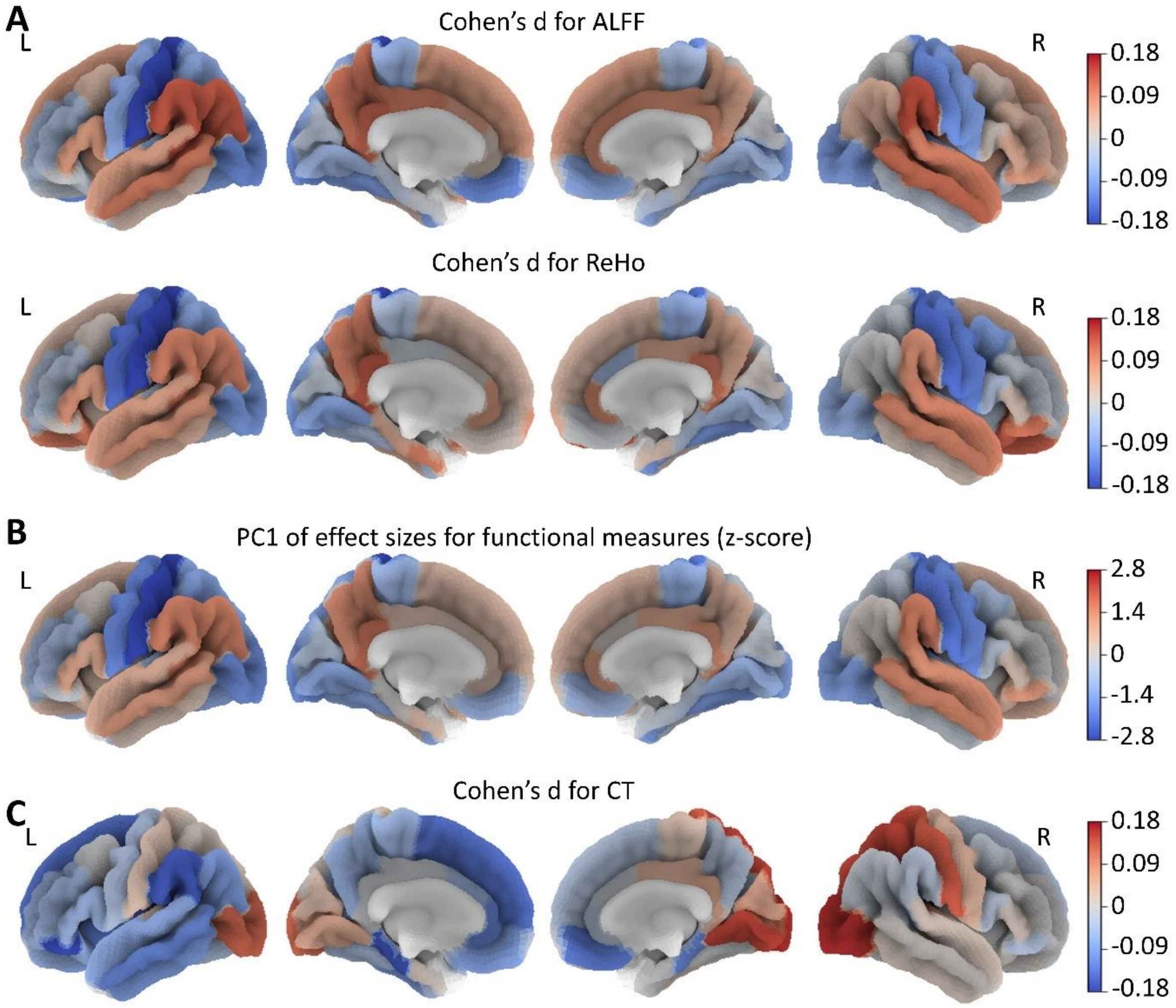


**Figure S4.** Case-control structural and functional brain differences in major depressive disorder (MDD) based on UK Biobank. **(A)** Differences (Cohen's d) for functional brain measures including amplitude of low-frequency fluctuation (ALFF) and regional homogeneity (ReHo); **(B)** The first principle component of effect sizes for functional measures (funcPC1) (Z-score normalization); **(C)** Differences (Cohen's d) for gray matter volume (GMV).

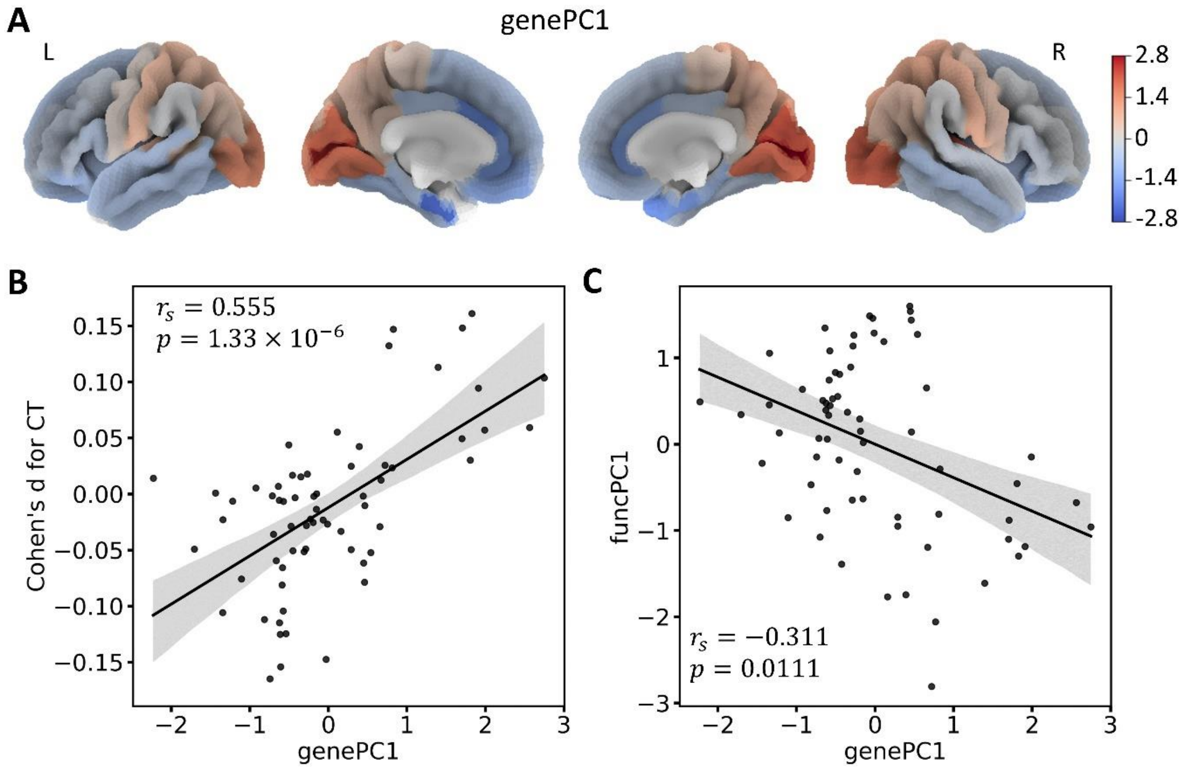


**Figure S5**. Relations of gene expression with case-control differences in UK Biobank. **(A)** The first principal component of gene expression (genePC1); **(B)** Relationships of genePC1 with cortical thickness (CT) differences; **(C)** Relationships of genePC1 with the first principal component of effect sizes for amplitude of low-frequency fluctuation (ALFF) and regional homogeneity (ReHo) (funcPC1).

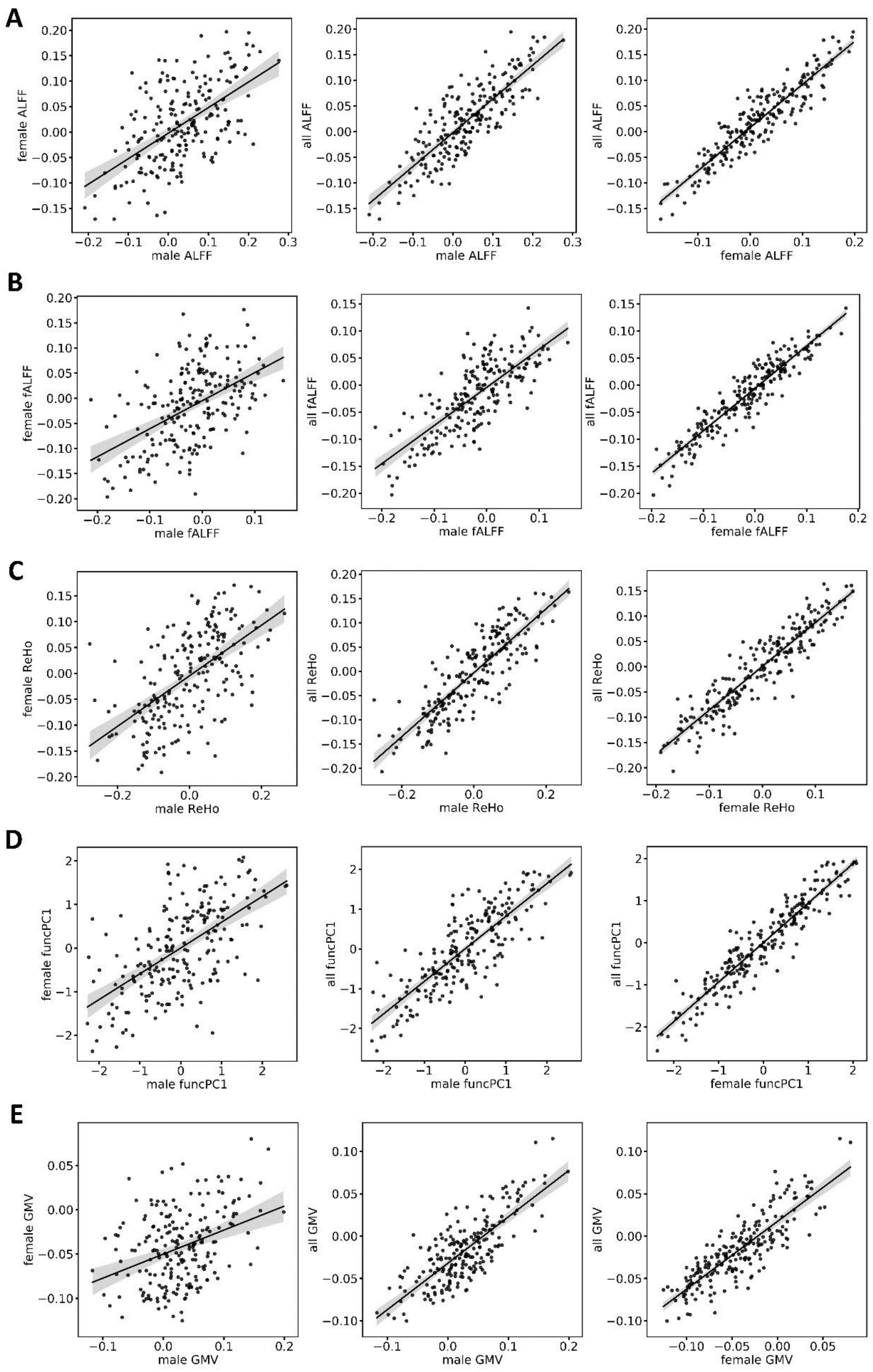


**Figure S6**. The relationships of structural and functional brain abnormalities observed in the male or female group with the abnormalities observed in whole dataset. **(A)** Amplitude of low-frequency fluctuation (ALFF); **(B)** Fractional ALFF (fALFF); **(C)** Regional homogeneity (ReHo); **(D)** The first principle component of effect sizes for functional measures (funcPC1); **(E)** Gray matter volume (GMV).

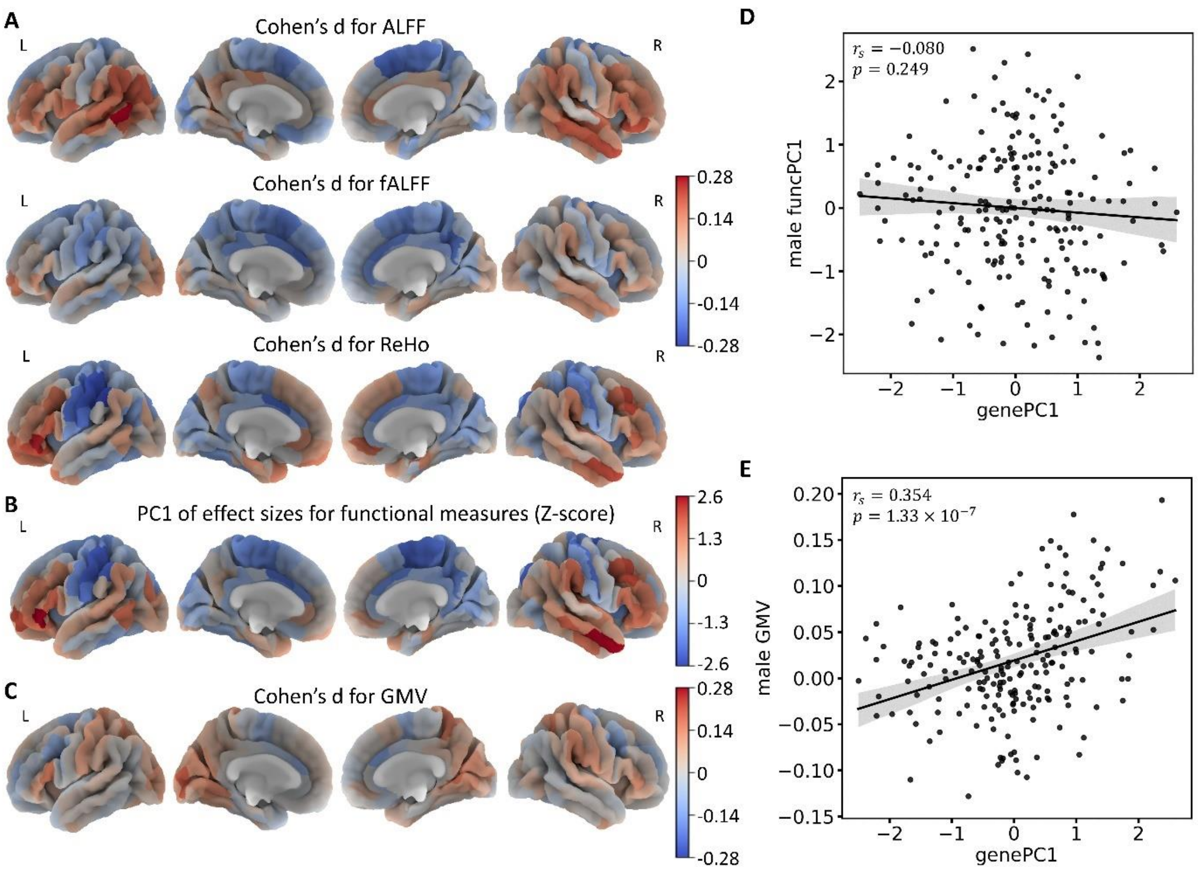


**Figure S7**. Relations of gene expression with case-control differences in the male group. **(A)** Differences (Cohen's d) for functional brain measures including amplitude of low-frequency fluctuation (ALFF), fractional ALFF (fALFF), and regional homogeneity (ReHo); **(B)** The first principle component of effect sizes for three functional measures (funcPC1) (Z-score normalization); **(C)** Differences **(**Cohen's d) for gray matter volume (GMV); **(D)** The relationship of first principal component of gene expression (genePC1) with the funcPC1; **(E)** The relationship of genePC1 with the GMV differences.

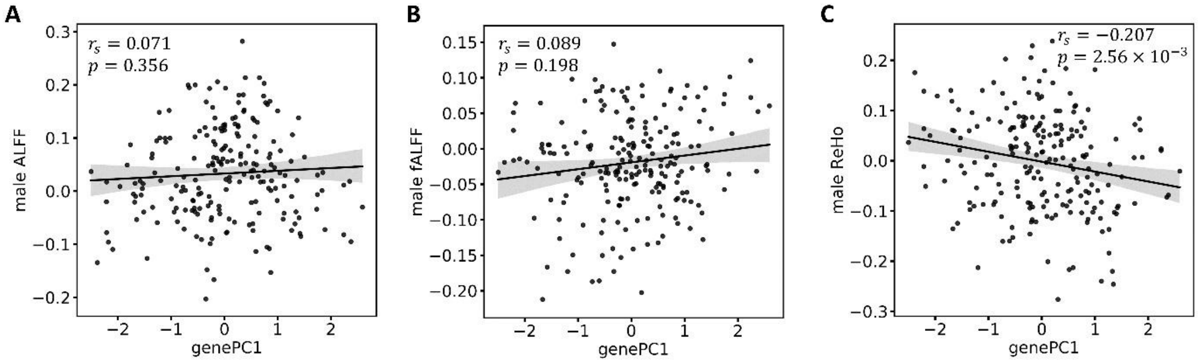


**Figure S8.** Relations of gene expression with three functional brain differences in the male group. **(A)** Amplitude of low-frequency fluctuation (ALFF); **(B)** Fractional ALFF (fALFF); **(C)** Regional homogeneity (ReHo).

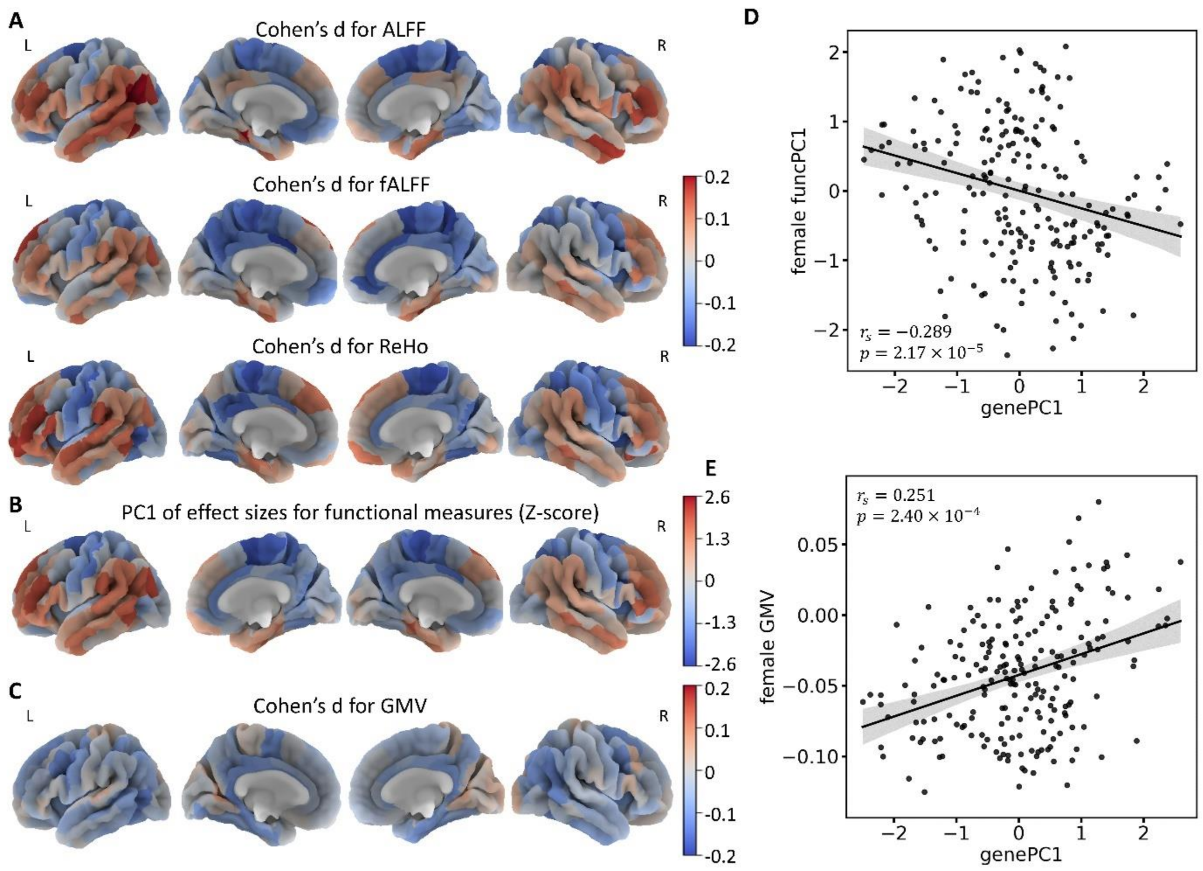


**Figure S9.** Relations of gene expression with case-control differences in the female group. **(A)** Differences (Cohen's d) for functional brain measures including amplitude of low-frequency fluctuation (ALFF), fractional ALFF (fALFF), and regional homogeneity (ReHo); **(B)** The first principle component of effect sizes for three functional measures (funcPC1) (Z-score normalization); **(C)** Differences **(**Cohen's d) for gray matter volume (GMV); **(D)** The relationship of first principal component of gene expression (genePC1) with the funcPC1; **(E)** The relationship of genePC1 with the GMV differences.

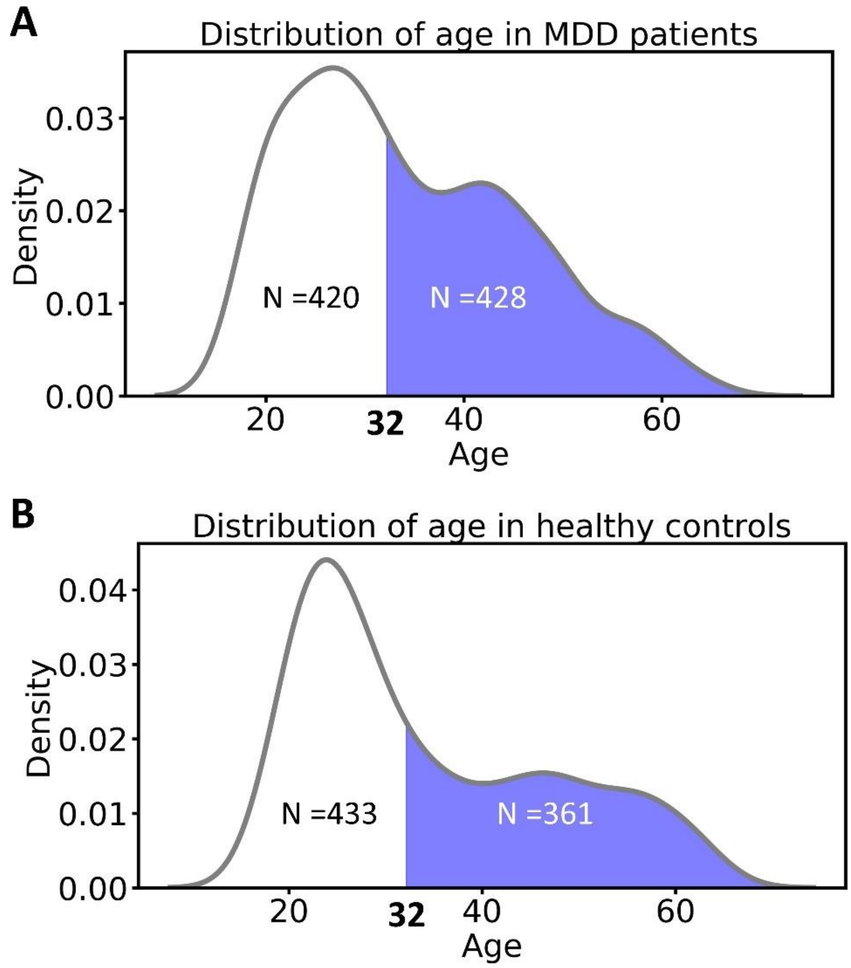


**Figure S10.** The distribution of age. **(A)** Major depressive disorder (MDD) patients; **(B)** Healthy controls.

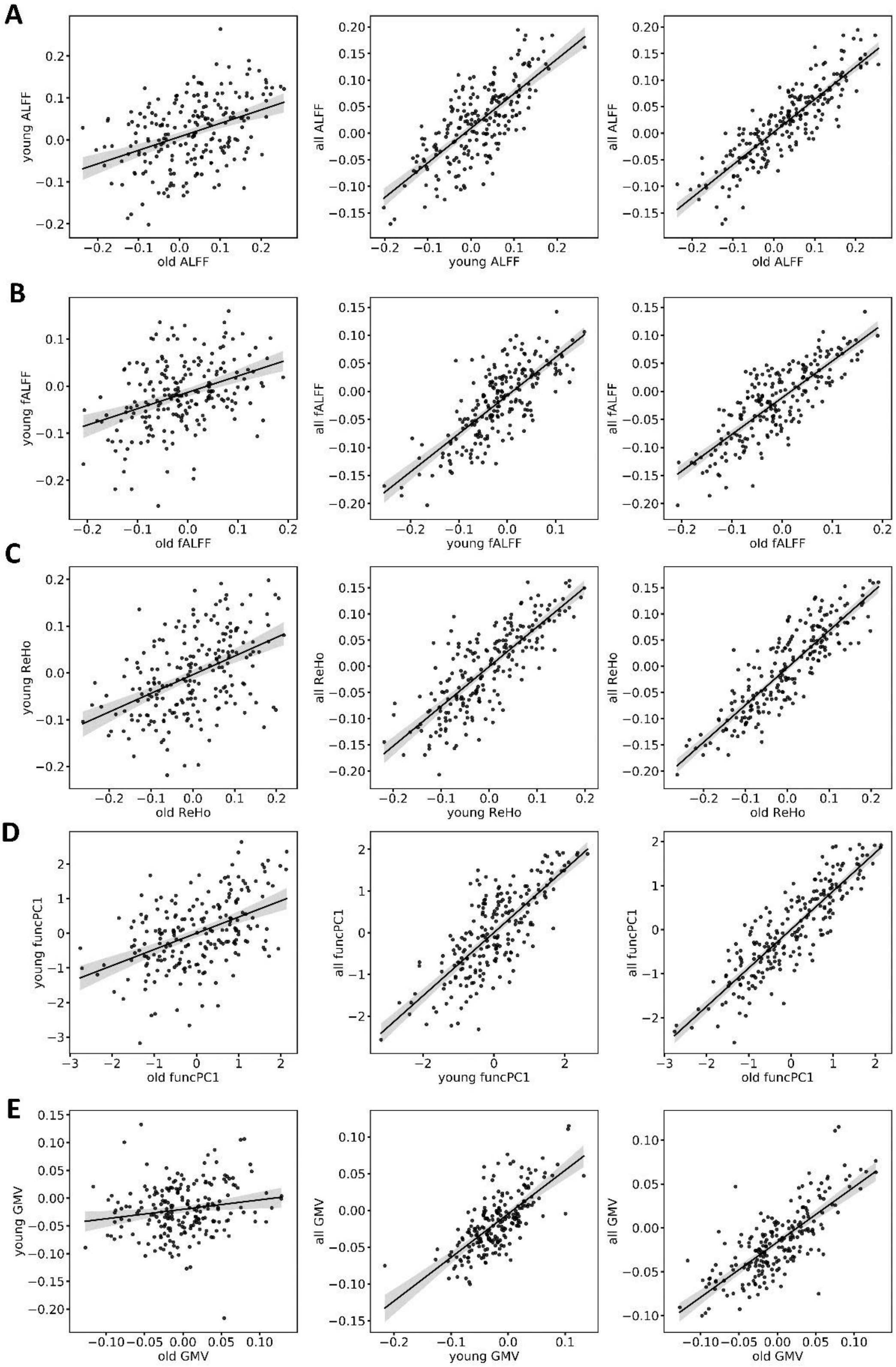


**Figure S11**. The relationships of structural and functional brain abnormalities observed in the older or younger group with the abnormalities observed in whole dataset. **(A)** Amplitude of low-frequency fluctuation (ALFF); **(B)** Fractional ALFF (fALFF); **(C)** Regional homogeneity (ReHo); **(D)** The first principle component of effect sizes for functional measures (funcPC1); **(E)** Gray matter volume (GMV).

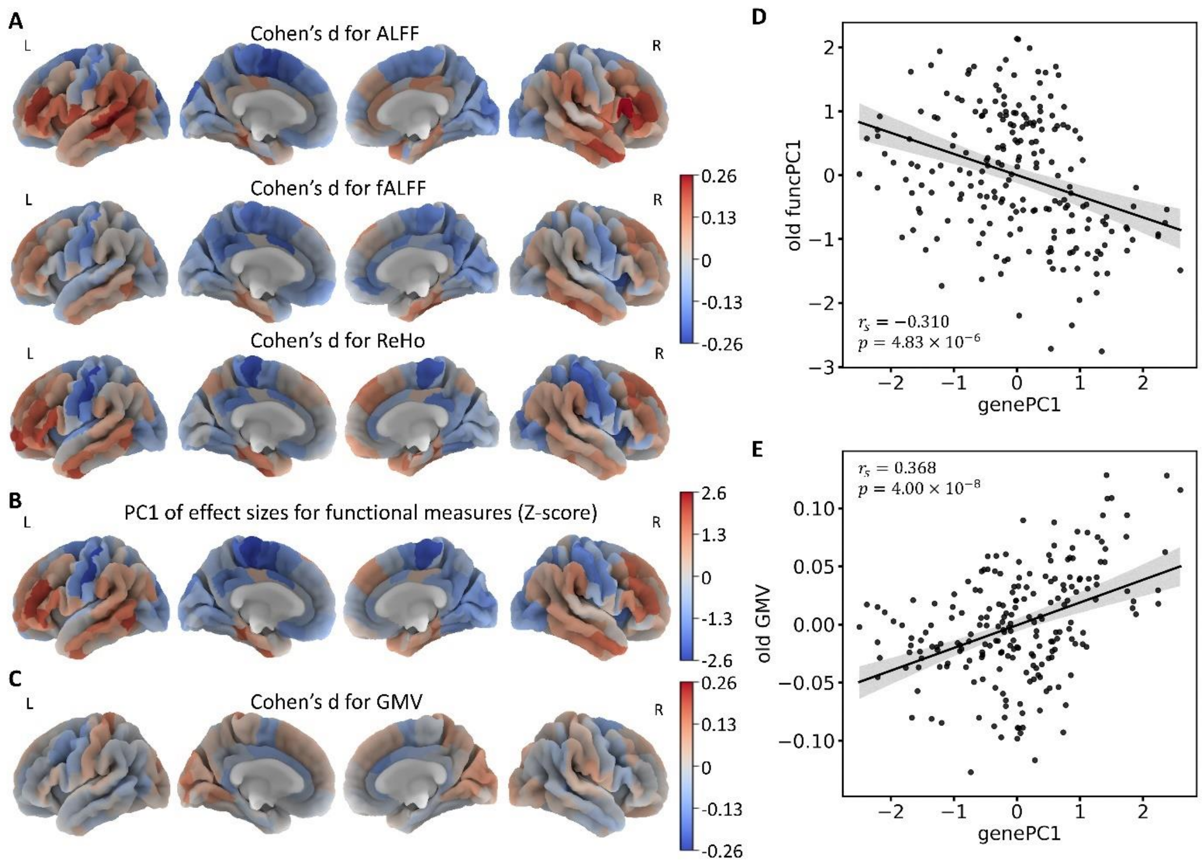


**Figure S12.** Relations of gene expression with case-control differences in the older group. **(A)** Differences (Cohen's d) for functional brain measures including amplitude of low-frequency fluctuation (ALFF), fractional ALFF (fALFF), and regional homogeneity (ReHo); **(B)** The first principle component of effect sizes for three functional measures (funcPC1) (Z-score normalization); **(C)** Differences **(**Cohen's d) for gray matter volume (GMV); **(D)** The relationship of first principal component of gene expression (genePC1) with the funcPC1; **(E)** The relationship of genePC1 with the GMV differences.

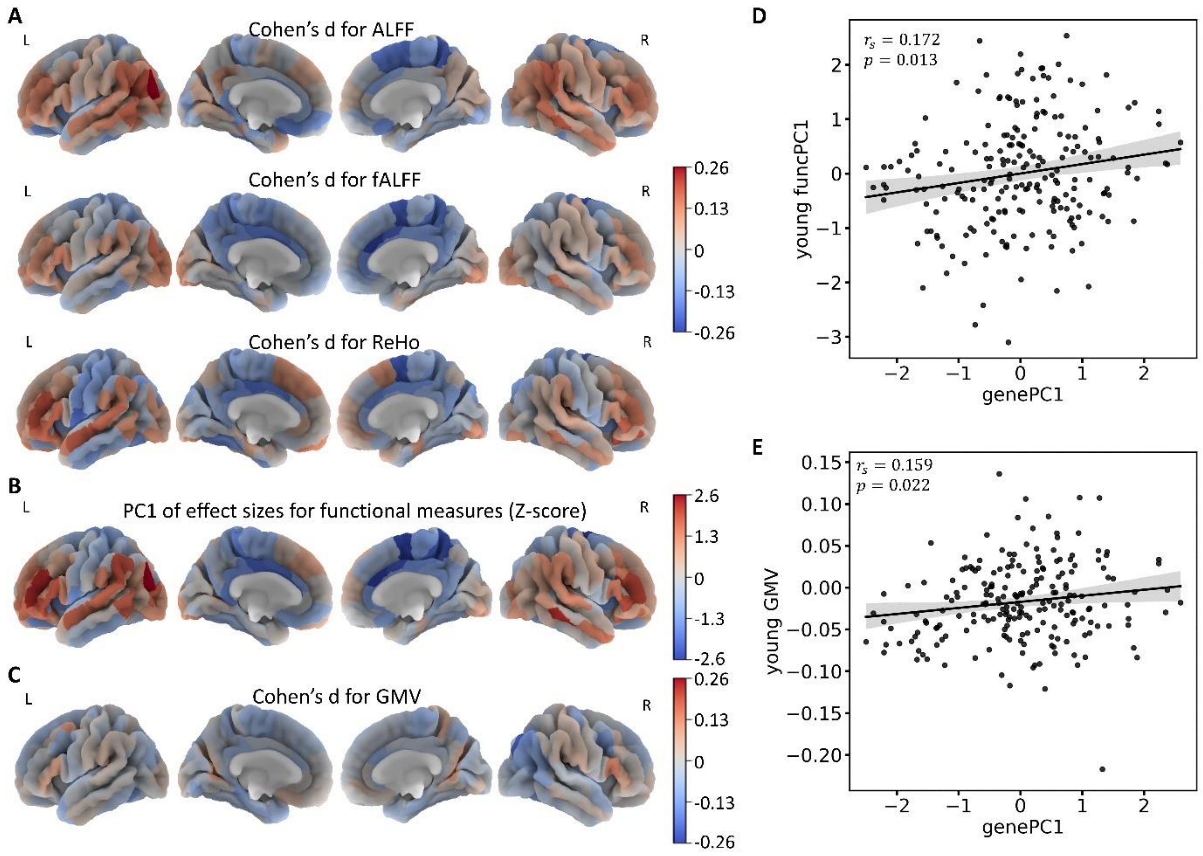


**Figure S13**. Relations of gene expression with case-control differences in the younger group. **(A)** Differences (Cohen's d) for functional brain measures including amplitude of low-frequency fluctuation (ALFF), fractional ALFF (fALFF), and regional homogeneity (ReHo); **(B)** The first principle component of effect sizes for three functional measures (funcPC1) (Z-score normalization); **(C)** Differences (Cohen's d) for gray matter volume (GMV); **(D)** The relationship of first principal component of gene expression (genePC1) with the funcPC1; **(E)** The relationship of genePC1 with the GMV differences.

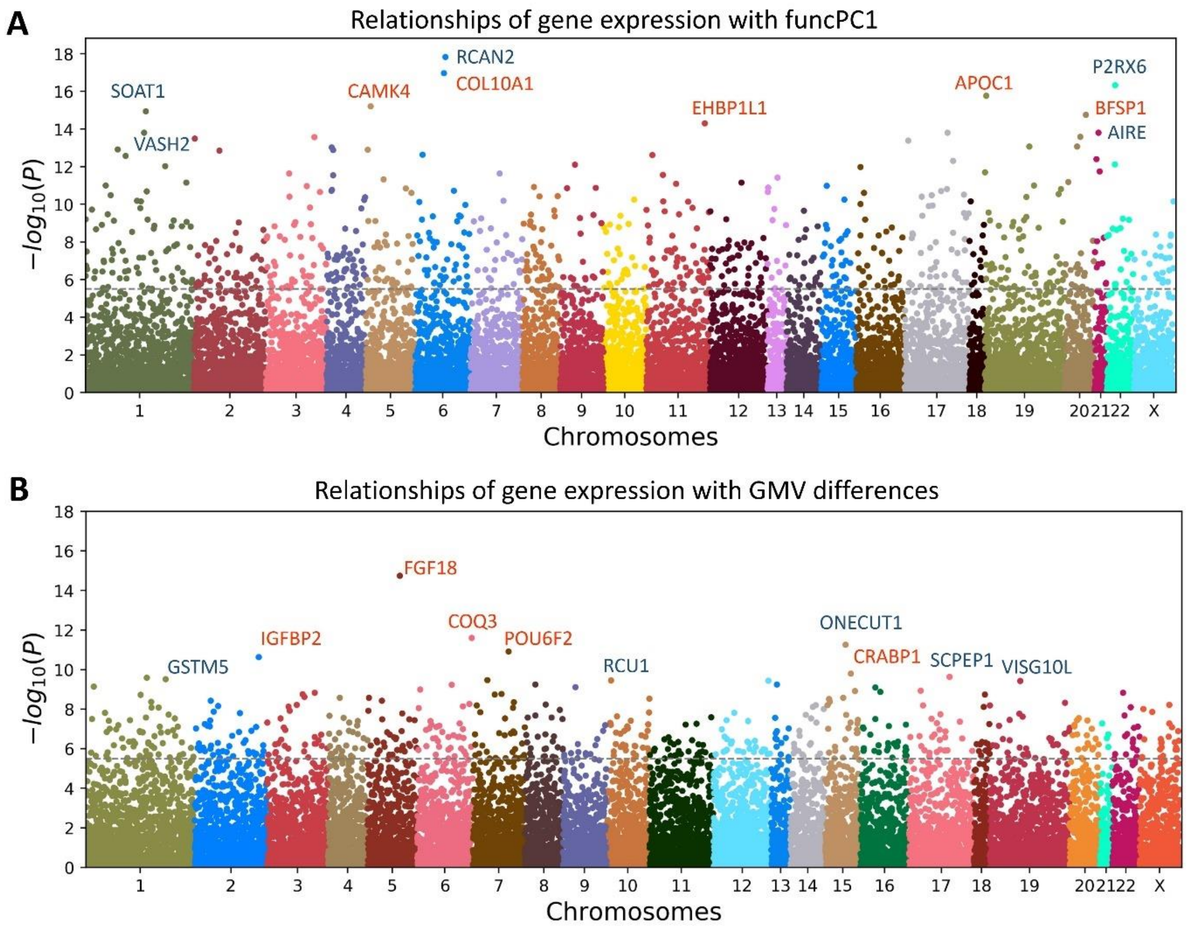


**Figure S14**. Manhattan plots showing associations between gene expression and cortical differences. **(A)** The first principal component of effect sizes for three major functional measures (funcPC1); **(B)** Gray matter volume (GMV) differences. X axis shows the chromosomes, and Y axis shows the $-\log_{10}(p)$ value, which indicates the significance of the association of the gene expression with cortical differences. The horizontal dotted line indicates the significance threshold after correcting for multiple testing.

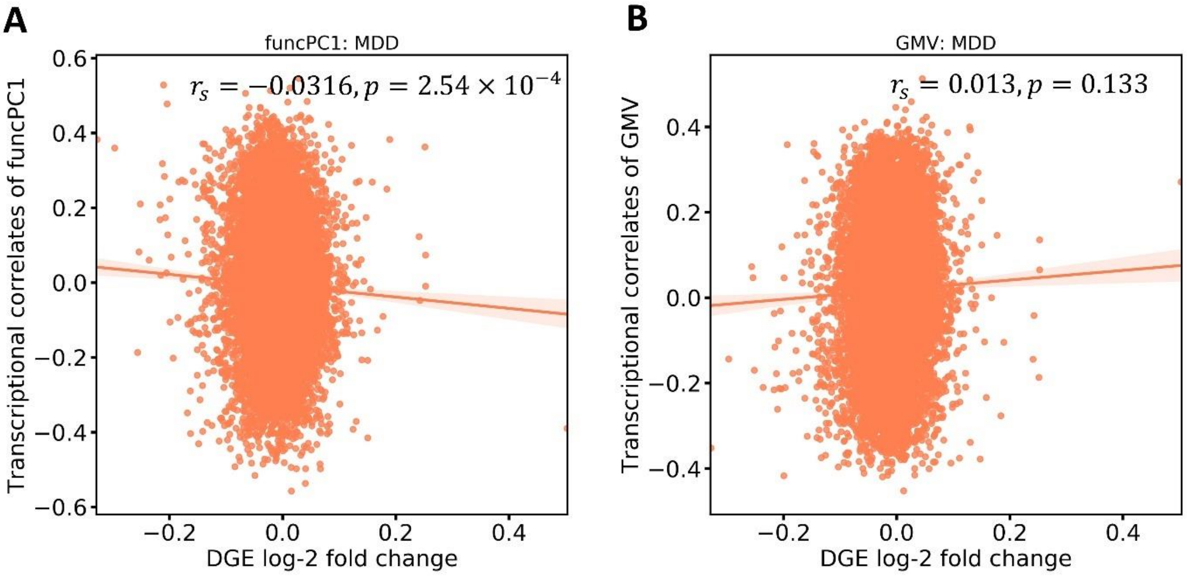


**Figure S15.** The correlations of the transcriptional correlates of brain abnormities with differential gene expression (DGE) values for major depressive disorder (MDD) when all genes are individually included. The funcPC1 is the first principal component of effect sizes for functional brain measures, and GMV indicates the gray matter volume.

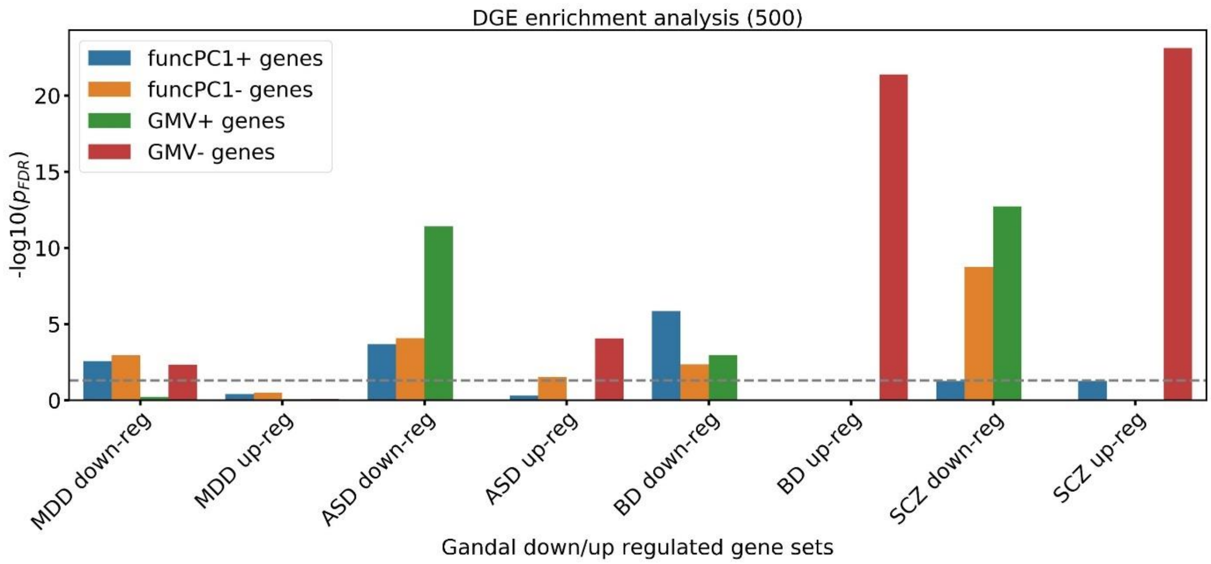


**Figure S16.** Enrichment analysis for 500 up- and down-regulated genes for psychiatric disorders. $p_{\mathrm{FDR}}$ is the adjusted $p$ value after FDR multiple testing correction. FuncPC1 is the first principal component of effect sizes for functional brain measures, and GMV indicates gray matter volume. Genes positively and negatively related to funcPC1 and GMV differences are defined as funcPC1+, funcPC1- genes, GMV+, and GMV- genes. Major depressive disorder, MDD; autism spectrum disorder, ASD; bipolar disorder, BP; schizophrenia, SCZ.

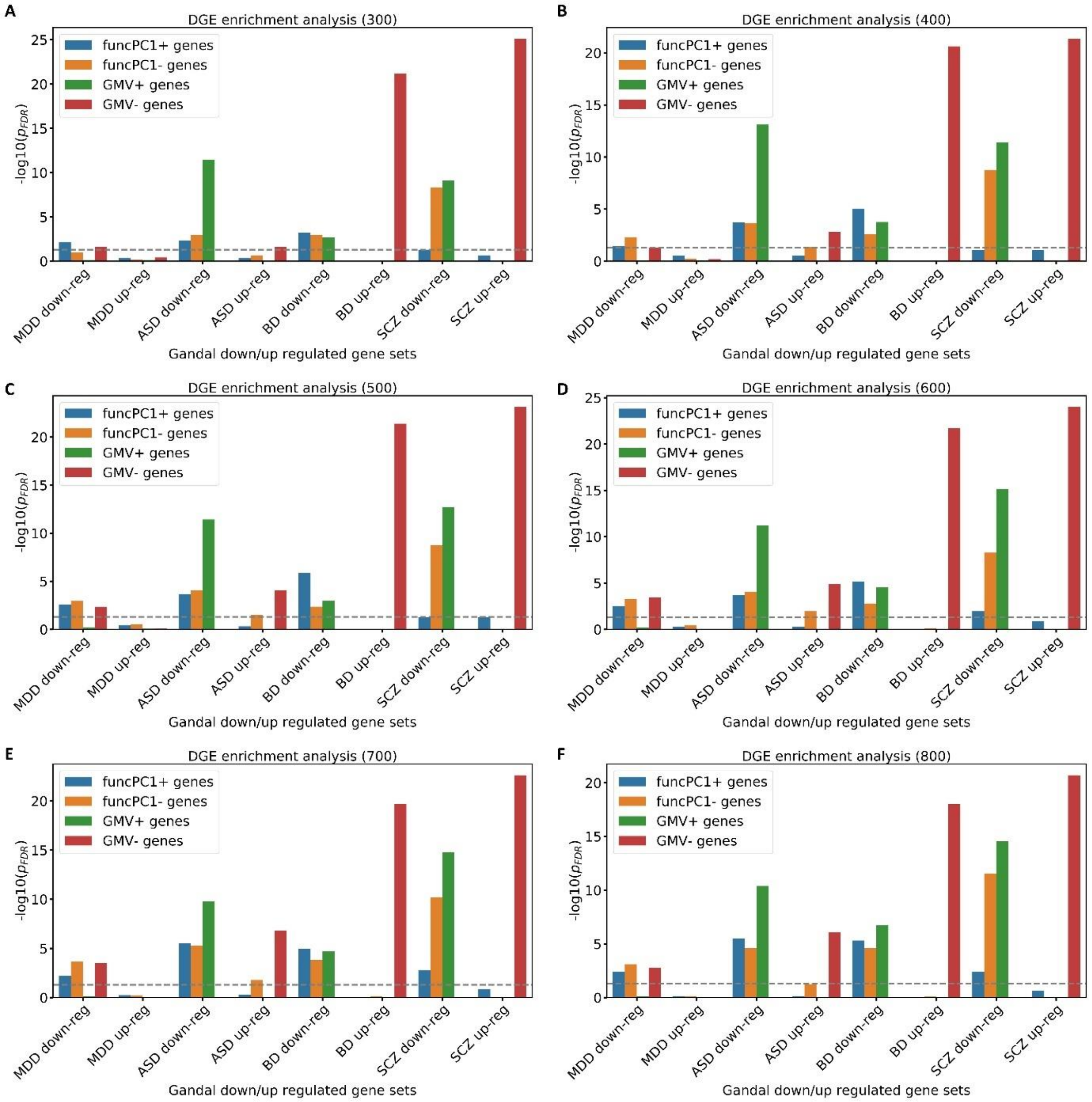


**Figure S17**. Enrichment analysis for up- and down-regulated genes at different thresholds (300-800) based on differential gene expression (DGE) analysis in psychiatric disorders. **(A)** 300; **(B)** 400; **(C)** 500; **(D)** 600; **(E)** 700; **(F)** 800. $p_{FDR}$ is the adjusted *p* value after FDR multiple testing correction. FuncPC1 is the first principal component of effect sizes for functional brain measures, and GMV indicates the gray matter volume. Genes positively and negatively related to funcPC1 and GMV differences are defined as funcPC1+, funcPC1- genes, GMV+, and GMV- genes. Major depressive disorder (MDD), autism spectrum disorder (ASD), bipolar disorder (BP), and schizophrenia (SCZ).

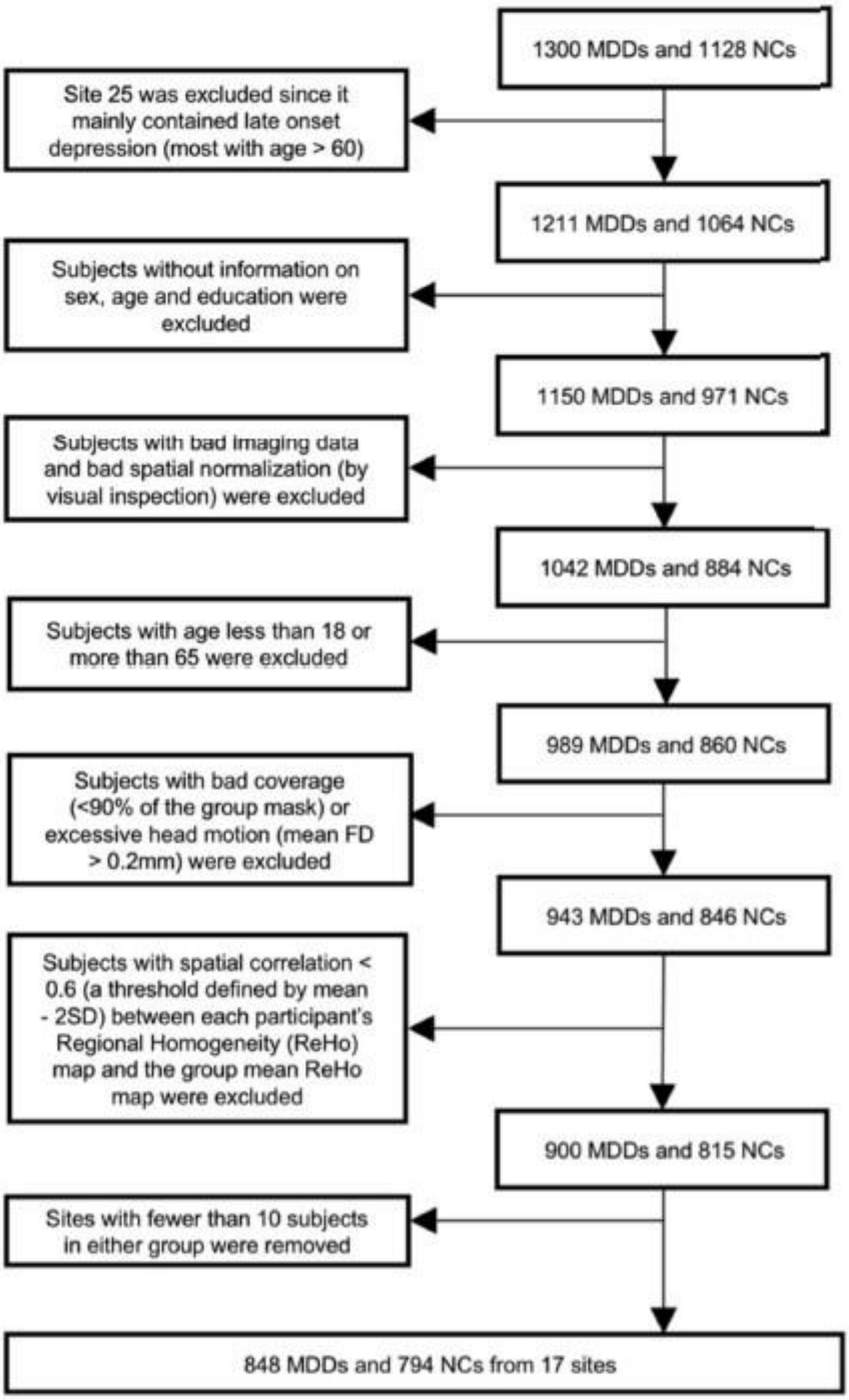


**Figure S18**. Sample selection of REST-meta-MDD dataset copied from the study by Yan et al..[6] (The open access article has been distributed under Creative Commons Attribution-NonCommercial-NoDerivatives License 4.0 (CC BY-NC-ND).)

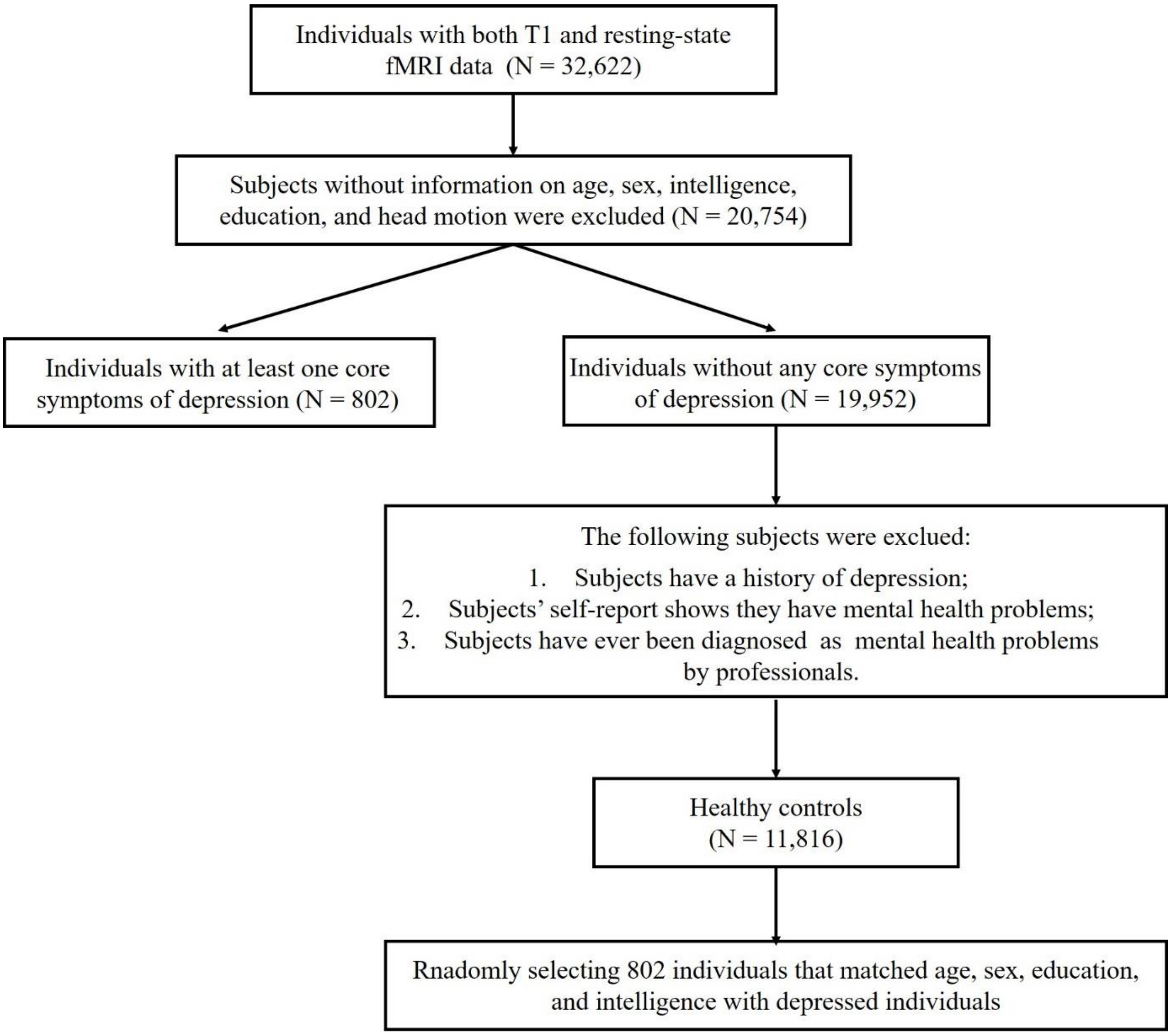


**Figure S19**. Sample selection of UK Biobank dataset.

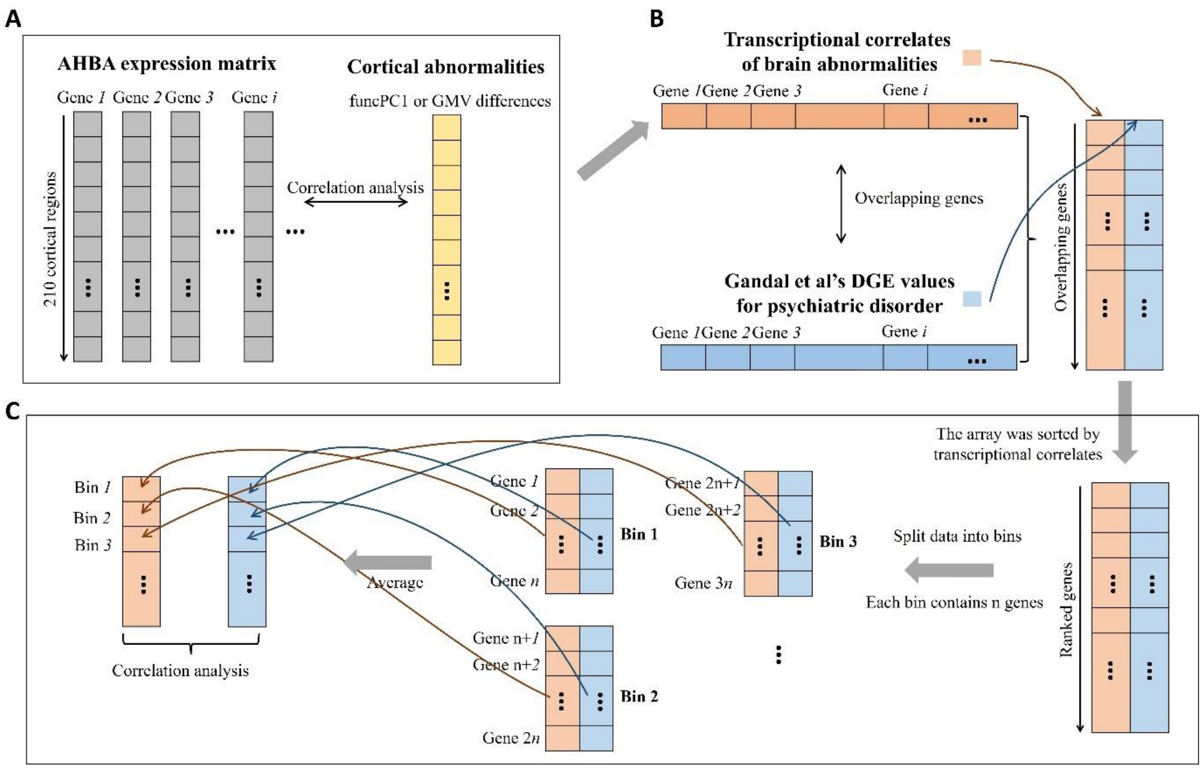


**Figure S20.** The flow chart of bin-based correlation analysis. **(A)** Transcriptional correlates of brain abnormalities; **(B)** Overlapping genes between AHBA and the study by Gandal et al.; **(C)** Clustering overlapping genes into 100 bins.

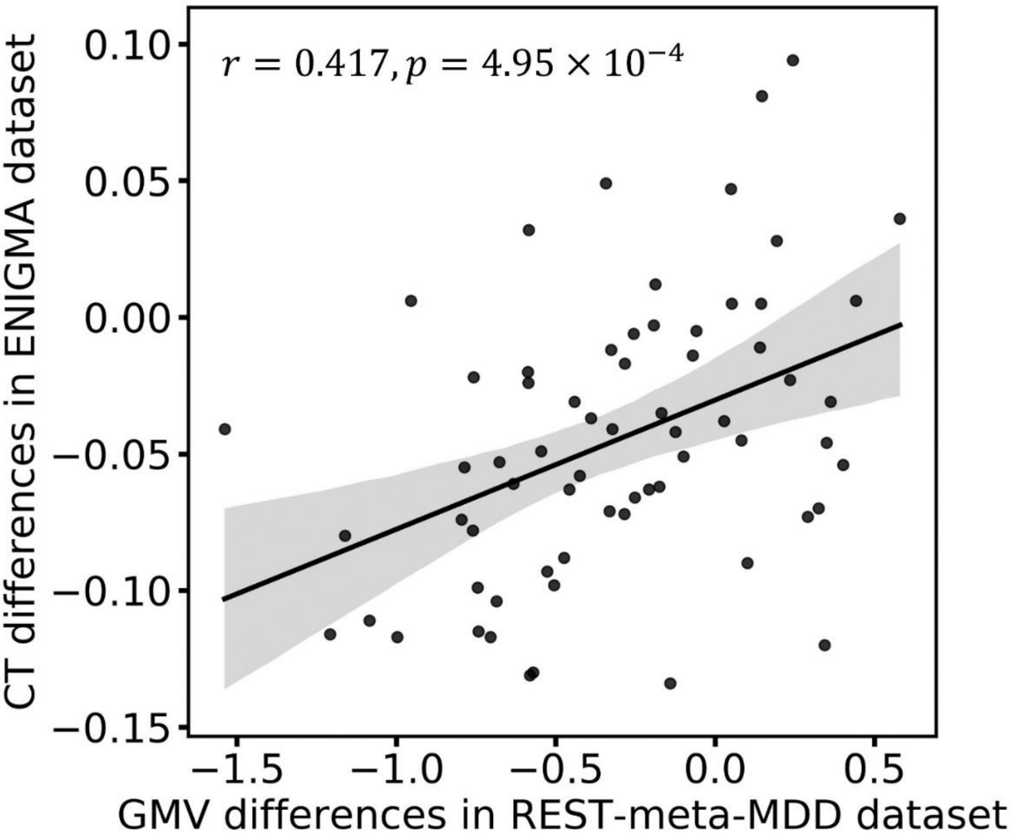


**Figure S21**. The relationship of structural brain abnormalities between REST-meta-MDD and ENIGMA dataset.

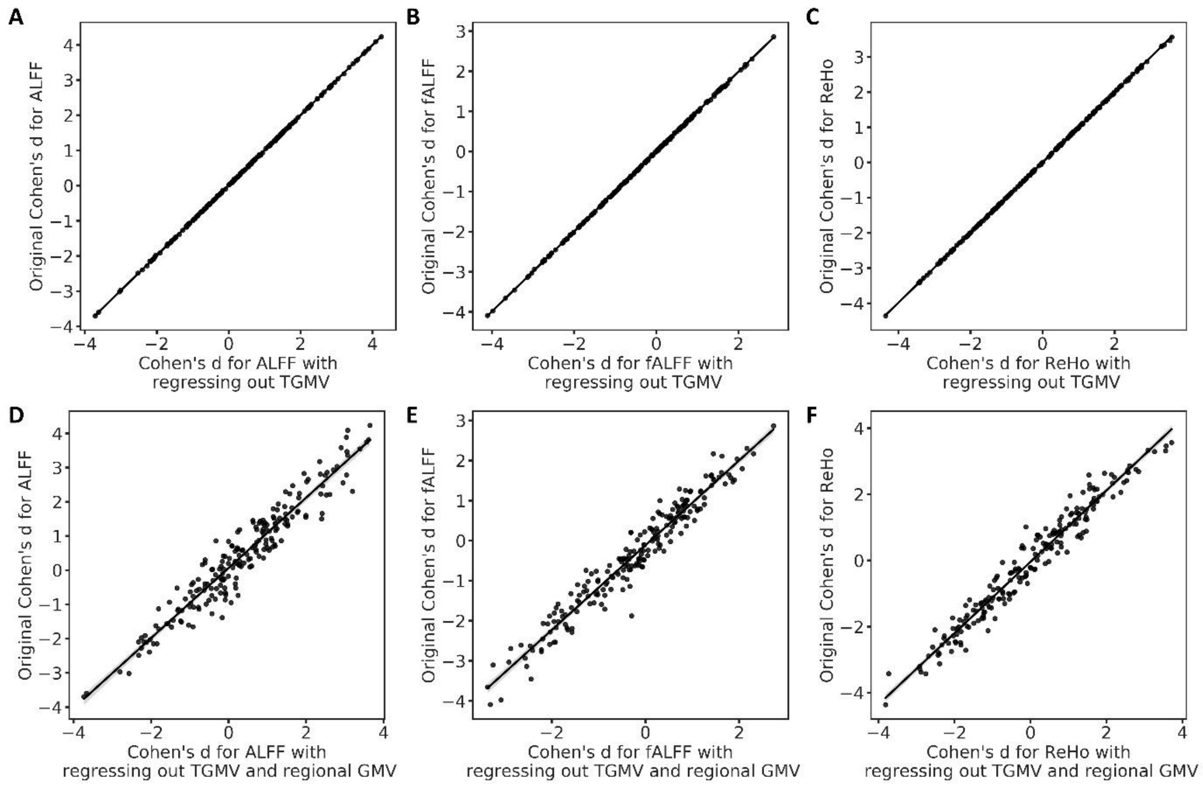


**Figure S22.** The effects of gray matter volume (GMV) on case-control functional brain differences. **(A)** Amplitude of low-frequency fluctuation (ALFF) corrected for total GMV (TGMV); **(B)** Fractional ALFF (fALFF) corrected for TGMV; **(C)** Regional homogeneity (ReHo) corrected for TGMV; **(D)** ALFF corrected for TGMV and regional GMV; **(E)** fALFF corrected for TGMV and regional GMV; **(F)** ReHo corrected for TGMV and regional GMV.

## References

[1] C. Bycroft, C. Freeman, D. Petkova, G. Band, L. T. Elliott, K. Sharp, A. Motyer, D. Vukcevic, O. Delaneau, J. O'Connell, A. Cortes, S. Welsh, A. Young, M. Effingham, G. McVean, S. Leslie, N. Allen, P. Donnelly, J. Marchini, *Nature* **2018**, 562(7726), 203-209.

[2] a) D. J. Smith, B. I. Nicholl, B. Cullen, D. Martin, Z. Ul-Haq, J. Evans, J. M. R. Gill, B. Roberts, J. Gallacher, D. Mackay, M. Hotopf, I. Deary, N. Craddock, J. P. Pell, *PLoS One* **2013**, 8(11), e75362; b) D. M. Howard, M. J. Adams, M. Shirali, T.-K. Clarke, R. E. Marioni, G. Davies, J. R. I. Coleman, C. Alloza, X. Shen, M. C. Barbu, E. M. Wigmore, J. Gibson, S. P. Hagenaars, C. M. Lewis, J. Ward, D. J. Smith, P. F. Sullivan, C. S. Haley, G. Breen, I. J. Deary, A. M. McIntosh, *Nat. Commun*. **2018**, 91(9), 1–10.

[3] R. S. Desikan, F. Ségonne, B. Fischl, B. T. Quinn, B. C. Dickerson, D. Blacker, R. L. Buckner, A. M. Dale, R. P. Maguire, B. T. Hyman, M. S. Albert, R. J. Killiany, *Neuroimage* **2006**, 31(3), 968-980.

[4] K. Xu, Y. Liu, Y. Zhan, J. Ren, T. Jiang, *Front. Neuroinform*. **2018**, 12, 52.

[5] L. Schmaal, D. P. Hibar, P. G. Sämann, G. B. Hall, B. T. Baune, N. Jahanshad, J. W. Cheung, T. G. M. Van Erp, D. Bos, M. A. Ikram, M. W. Vernooij, W. J. Niessen, H. Tiemeier, A. Hofman, K. Wittfeld, H. J. Grabe, D. Janowitz, R. Bülow, M. Selonke, H. Völzke, D. Grotegerd, U. Dannlowski, V. Arolt, N. Opel, W. Heindel, H. Kugel, D. Hoehn, M. Czisch, B. Couvy-Duchesne, M. E. Rentería, L. T. Strike, M. J. Wright, N. T. Mills, G. I. De Zubicaray, K. L. McMahon, S. E. Medland, N. G. Martin, N. A. Gillespie, R. Goya-Maldonado, O. Gruber, B. Krämer, S. N. Hatton, J. Lagopoulos, I. B. Hickie, T. Frodl, A. Carballedo, E. M. Frey, L. S. Van Velzen, B. W. J. H. Penninx, M. J. Van Tol, N. J. Van der Wee, C. G. Davey, B. J. Harrison, B. Mwangi, B. Cao, J. C. Soares, I. M. Veer, H. Walter, D. Schoepf, B. Zurowski, C. Konrad, E. Schramm, C. Normann, K. Schnell, M. D. Sacchet, I. H. Gotlib, G. M. MacQueen, B. R. Godlewska, T. Nickson, A. M. McIntosh, M. Papmeyer, H. C. Whalley, J. Hall, J. E. Sussmann, M. Li, M. Walter, L. Aftanas, I. Brack, N. A. Bokhan, P. M. Thompson, D. J. Veltman, *Mol. Psychiatry* **2017**, 22(6), 900-909.

[6] C. G. Yan, X. Chen, L. Li, F. X. Castellanos, T. J. Bai, Q. J. Bo, J. Cao, G. M. Chen, N. X. Chen, W. Chen, C. Cheng, Y. Q. Cheng, X. L. Cui, J. Duan, Y. R. Fang, Q. Y. Gong, W. Bin Guo, Z. H. Hou, L. Hu, L. Kuang, F. Li, K. M. Li, T. Li, Y. S. Liu, Z. N. Liu, Y. C. Long, Q. H. Luo, H. Q. Meng, D. H. Peng, H. T. Qiu, J. Qiu, Y. Di Shen, Y. S. Shi, C. Y. Wang, F. Wang, K. Wang, L. Wang, X. Wang, Y. Wang, X. P. Wu, X. R. Wu, C. M. Xie, G. R. Xie, H. Y. Xie, P. Xie, X. F. Xu, H. Yang, J. Yang, J. S. Yao, S. Q. Yao, Y. Y. Yin, Y. G. Yuan, A. X. Zhang, H. Zhang, K. R. Zhang, L. Zhang, Z. J. Zhang, R. B. Zhou, Y. T. Zhou, J. J. Zhu, C. J. Zou, T. M. Si, X. N. Zuo, J. P. Zhao, Y. F. Zang, *Proc. Natl. Acad. Sci.* **2019**, 116(18), 9078-9083.
